# Molecular and Computational Strategies to Increase the Efficiency of CRISPR-Based Techniques

**DOI:** 10.3389/fpls.2022.868027

**Published:** 2022-05-31

**Authors:** Lucia Mattiello, Mark Rütgers, Maria Fernanda Sua-Rojas, Rafael Tavares, José Sérgio Soares, Kevin Begcy, Marcelo Menossi

**Affiliations:** ^1^Department of Genetics, Evolution, Microbiology and Immunology, Institute of Biology, State University of Campinas (UNICAMP), Campinas, Brazil; ^2^Cell and Developmental Biology, John Innes Centre, Norwich, United Kingdom; ^3^Environmental Horticulture Department, University of Florida, Gainesville, FL, United States

**Keywords:** CRISPR/Cas9, base editing, prime editing, genome editing efficiency, DNA-free CRISPR/Cas

## Abstract

The prokaryote-derived Clustered Regularly Interspaced Palindromic Repeats (CRISPR)/Cas mediated gene editing tools have revolutionized our ability to precisely manipulate specific genome sequences in plants and animals. The simplicity, precision, affordability, and robustness of this technology have allowed a myriad of genomes from a diverse group of plant species to be successfully edited. Even though CRISPR/Cas, base editing, and prime editing technologies have been rapidly adopted and implemented in plants, their editing efficiency rate and specificity varies greatly. In this review, we provide a critical overview of the recent advances in CRISPR/Cas9-derived technologies and their implications on enhancing editing efficiency. We highlight the major efforts of engineering Cas9, Cas12a, Cas12b, and Cas12f proteins aiming to improve their efficiencies. We also provide a perspective on the global future of agriculturally based products using DNA-free CRISPR/Cas techniques. The improvement of CRISPR-based technologies efficiency will enable the implementation of genome editing tools in a variety of crop plants, as well as accelerate progress in basic research and molecular breeding.

## Introduction

The capability of creating genetic variation has always been the key for crop improvement (Griggs et al., 2013). While this is traditionally achieved through conventional plant breeding or random mutagenesis induced by ionizing radiation and chemical mutagens, nowadays, agricultural biotechnology has entered a new era of nucleotide-scale precision (Jung and Till, 2021).

Site-directed nucleases are used to produce single- or double-strand breaks at specific DNA target sites. These DNA breaks stimulate either the stochastic non-homologous end joining (NHEJ) or homology directed repair (HDR) pathway, resulting in diverse outcomes such as site-directed mutagenesis, gene replacement, and nucleotide insertions or deletions (Kumar and Jain, 2015). While hybrid enzymes, like meganucleases, Zinc Finger Nuclease (ZNF), and Transcription activator-like Effector Nucleases (TALEN), have been successfully used for genome editing in plants (Curtin et al., 2012; Osakabe and Osakabe, 2015) the clustered regularly interspaced short palindromic repeat (CRISPR)/CRISPR-associated protein (Cas) system is the new gold standard, because it is more versatile, less expensive, simpler and more reliable (Belhaj et al., 2013).

CRISPR/Cas9 was first discovered as a bacterial and archaeal defense system against transmissible elements from virus or exogenous DNA (Mojica et al., 2000; Mojica and Rodriguez-Valera, 2016). The rapid adoption of CRISPR/Cas9 technique originated from *Streptococcus pyogenes*, enabled the initial application as a genome editing tool in plants and animals (Makarova et al., 2011a,b; Hsu et al., 2014).

Even though CRISPR/Cas9 technology has been rapidly adopted and successfully implemented in plants, its gene editing efficiency rate varied greatly (Howells et al., 2018). Recently, several robust technical improvements have emerged based on the necessity to improve efficiency and specificity. This review focuses on highlighting the different computational and experimental base and prime editing strategies developed to increase the efficiency of CRISPR/Cas-based genome editing systems. We first describe recent advances in deep learning tools used during CRISPR/Cas constructs design. We then present a comprehensive overview of the recently developed Cas9 and prime editing (PE) tools and their implications on enhancing editing efficiency. We also provide a perspective on the global future of agricultural based products using DNA-free CRISPR/Cas techniques. The advances we discuss here point to several venues of experiencing that are paving the way to increase the efficiency in producing homozygous edits, the occurrence of off target editing, the broadening of possibilities of target sites in the genome and to enhance homologous recombination. Addressing these key issues will have a major impact on the way we can modify the genome of crops, leveraging the efforts to a sustainable agriculture and preparing crops to a changing environment.

## The CRISPR/Cas9 System

### Deep-Learning Strategies to Increase CRISPR Cleavage Efficiency

The rapid progress of experimental procedures implementing CRISPR/Cas technology in plants over the past decade has been accompanied by equally impressive advances in the computational methods for gRNA design and off target prediction (Wang et al., 2017b, 2020b; Lowder et al., 2018; Hajiahmadi et al., 2019). As genetic transformation methods and CRISPR implementation grew, the emerging algorithm developments for accurate target prediction revealed increasingly complex facets of the underlying biology, from sequencing composition to epigenetic regulation (Wang et al., 2019; Yan et al., 2020). At the same time, rapid growth has forced constant reevaluation of the underlying algorithms and statistical models used by these computational tools when implemented in CRISPR studies (Sledzinski et al., 2020).

The development of computational tools has become essential in the process of single guide RNA (sgRNAs) design. However, one of the major challenges of the CRISPR system is to precisely predict the sgRNA on-target knock-in or knock-out efficacy, while avoiding undesired cuts to similar DNA sequences in the target genome creating off-targets. In plants as well as in other organisms, a large difference in cleavage efficiency has been observed, which suggests that several factors regulate the binding and cutting efficacy of the sgRNA-Cas complex (Raitskin et al., 2019; Ren et al., 2019b; Banakar et al., 2020; Wolabu et al., 2020; Grützner et al., 2021). Those factors include sequence composition, nucleotide position, GC content, chromatin accessibility, gene expression profile, RNA secondary structure, melting temperature and free energy (Kim et al., 2019; Safari et al., 2019; Xiang et al., 2021). Thus, a careful design of the sgRNA is essential for optimal Cas9 activity. It is critical to search for conserved domains, motifs, or regions within the target sequence among different genotypes or related species to make sure that there are no point mutations at the gRNA binding site or at the PAM site. A second important aspect to take into consideration is the presence of favorable nucleotides at the seed sequence (10 nucleotides closest to the PAM site; Doench et al., 2014; Xu et al., 2015). To date, the methods developed for sgRNA efficacy prediction and on-target identification can be classified into three major groups:

(1) Alignment-based methods: sgRNAs candidates are designed based on an alignment from the target sequence and the respective genome purely by locating the PAM sequence.
(2) Hypothesis-driven: sgRNAs efficiencies are scored empirically by compiling information related to the factors that impact the genome context.
(3) Machine and Deep learning-based: sgRNAs are predicted from a training model by considering different features.

Over the last couple of years, solid evidence has shown that hypothesis-driven and learning-based strategies outperform alignment-based strategies (Wang et al., 2020c; Yan et al., 2020; Zhang et al., 2020). However, the popularity gained by artificial intelligence has positioned learning-based methods as the leading strategies to increase efficiency efforts. Building reliable models to design highly efficient sgRNA using learning-based strategies largely depends on the critical factors that the models are built on. For instance, it is required a considerably large amount of data of sgRNAs designed from different platforms and their respective experimental efficiencies to build reliable models. In addition, experimental data on real quantification of off-target site prediction based on all the possible nucleotide mismatch loci at the whole genome level are limited (Zischewski et al., 2017). And finally, other genomic and epigenetic features that affect sgRNA remain unclear and could improve its efficacy.

Several machine and deep learning-based methods have been developed to predict CRISPR on-target activity. However, most of them use data sets exclusively for animal models (Chuai et al., 2018; Kim et al., 2019; Yan et al., 2020; Xiang et al., 2021). Even though animals and plants share genetic and epigenetic features, models specifically for plants are needed. Based on their statistical methods (Feng et al., 2017; Lin and Wong, 2018; Chen et al., 2019b; Wang et al., 2019, 2020c; Liu et al., 2020; Padilha et al., 2020; Yan et al., 2020; Zhang et al., 2020; O’Brien et al., 2021), most of the models developed can be roughly classified into six categories: (i) CNN: Convolution Neural Network; (ii) L1-Reg: L1-Regression; (iii) SVM: Support Vector Machine; (iv) RF: Random Forest; (v) GBRT: Gradient Boost Regression Tree; and (vi) SVM (C): using SVM to classify (+1 represents high activity, −1 represents low-activity).

Many of these statistical methods use algorithms to recognize targets based on the gRNA-DNA pairing. For instance, CRISPR-P, one of the most popular tools for sgRNA design in plants, supports sgRNA design for almost 50 plant species including gene annotation. In addition, it provides a sgRNA scoring system for on-target efficiency and off-target (Lei et al., 2014). Interestingly, it also supports various CRISPR-Cas systems including Cas12a (formerly Cpf1). Nevertheless, CRISPR-P allows uploading custom sequences and identifying sgRNAs if the genome of interest is not listed between the ones available. However, no off-target is available for that option (Liu et al., 2017a). Another interesting web-based tool, CRISPR-PLANT, calculates specificity of all gRNA spacer sequences based on both mismatches number and position in their alignments with other spacer sequences for any given sequence (Xie et al., 2014; Minkenberg et al., 2019). However, CRISPR-PLANT only allows searching for NGG-PAM sites. It would be interesting to have the possibility to upload genome sequences from other species, as implemented in CRISPR-P.

### Learning Cas9 Machines

Rational engineering of Cas9 nuclease has required conformational and mechanistic understanding of the CRISPR-Cas system, which has contributed greatly to push the boundaries of this technology. Currently, a vast repertoire of engineered or evolved SpCas9 variants (e.g., evoCas9, xCas9, HypaCas9, SpCas9-HF1, eSpCas9, and HiFi Cas9) have been created by rational design or directed evolution, altering catalytic function, PAM requirements and reducing off-target activity (for a detailed review on these Cas9 variants see Meaker et al., 2020). As such, the different combinations of key amino acid changes in the functional domains of Cas9 have improved its specificity, genome coverage and consequently, its efficiency. Nonetheless, despite remarkable progress, the existence of a guide-dependent trade-off between specificity and cleavage activity has been described among the panel of these novel variants (with the exception of xCas9), still leaving researchers puzzled by which Cas9 variants to use at a given locus (Schmid-Burgk et al., 2020).

Different features associated with gRNA activity (e.g., differences of nucleotide preference, and position-dependent nucleotide composition) between wild-type SpCas9 and these highly specific Cas9 proteins, explain to some extent the high dependence of these variants on the target sequences. Yet, far from meeting an ideal Cas9 (i.e., with high specificity and activity regardless of the guide RNA used), the use of techniques of relatively low-throughput and the lack of extensive comparisons among variants, due to an exceedingly costly task and labor-intensive, have limited novel biological insights.

These unprecedented challenges regarding the analysis and interpretation of Cas9 variants along with the astronomical numbers of theoretical targetable sequences (4^20^ = ca. 10^12^ molecules) have recently directed researchers to look at other alternatives to handle these massive amounts of data. Recently, for instance, an elegant bacterial system (termed Self-targeting sgRNA Library Screen—SLS) was developed to characterize these tailored Cas9 proteins, exploiting more than a million sequence libraries, wherein over 60 physicochemical parameters were considered (Tálas et al., 2021). Interestingly, the study identified various sequence features that positively or adversely impact on SpCas9-HF1 cleavage. This variant prefers a slightly higher GC-content in the middle region compared to other ones of the spacer. However, the presence of motifs corresponding or overlapping to the GTNAC sequence, also in the middle region (positions 10–14), affects its ability to form cleavage-competent conformation. Besides, it is noteworthy to mention that the SLS approach presented a robust prediction tool for mammalian cleavage activities too, which might open the use of a dozen SpCas9 variants.

Another recent high-throughput methodology that has been gaining a growing interest for decoding the variant activities dependency on target sequences is deep learning. Briefly, deep learning is a set of machine learning techniques based on stacked artificial neural network layers that can learn rich data representations from raw inputs through affine transformations and non-linear activation function (Xu et al., 2020c). Recently, using a combination of lentiviral libraries and DL-based computational models, Kim et al. (2019) have assembled an extensive comparison of SpCas9 variants, providing a helpful and general guide (Kim et al., 2020b). This online tool called DeepSpCas9variants allows users to select the most appropriate and effective variant to use at a given target sequence according to 20 most important features for predicting their activities. However, the greatest challenge will be to obtain the best identified Cas9 variants and optimize them for wet lab experiments.

Interestingly, CRISPRon, a recently developed tool, provides more accurate gRNA efficiency prediction outperform the existing tools developed so far (Xiang et al., 2021). When compared with DeepSpCas9variants, Azimuth, and DeepSpCas9, CRISPRon exceeded the performance showing a higher Spearman’s rho correlation (0.80; Xiang et al., 2021).

Given the Cas9 dependency of the protospacer DNA and the above-mentioned astronomical amount of possible permutations, it might seem unrealistic a library of Cas9 variants suitable for any possible composition of the target sequence. On the other hand, this raises the question of whether, in the near future, an ideal Cas9 suitable for all target DNA might be achievable. Fortunately, the field of deep learning for protein engineering is also moving rapidly and outperforming conventional methods. Besides, this approach has not yet been explored and used for the Cas9 protein structural diversity to identify good adaptive routes to higher fitness (Gao et al., 2020). Concomitant with that, the need of multiple mutated residues in Cas9 has been predicted to improve specificity and efficiency by changing the conformational dynamics and biophysical properties of the iCas9 at different stages of its activity (Palermo et al., 2017, 2018; Boyle et al., 2021; Wang et al., 2021). Mostly, these residues mediate the allosteric communication of Cas9 (i.e., flexibility of the three conformational stages) and the RNA/DNA heteroduplex to ensure the proper positioning of the catalytic site and the proofreading step (Yang et al., 2018). However, to find multiple mutations at one time is beyond the capacity of most screenings. In addition, the extreme epistatic interactions, so-called sign epistasis (i.e., the result of a mutation depends on the preceding mutations), in Cas9 variants may be a constraint on the path to an optimum fitness peak (Poelwijk et al., 2007; Romero and Arnold, 2009). Thereby, the use of conservative computational approaches such as UniRep (Alley et al., 2019), low-N (Biswas et al., 2021), and 1D convolutional neural network (CNN) is gaining much ground nowadays to the study of large-scale conformational changes of Cas9 and to accelerate engineered proteins (Orellana, 2019; Gao et al., 2020).

## Increasing the PAM Sequence Scope

*Streptococcus pyogenes* Cas9 is the most widely used protein in the CRISPR/Cas system for genome editing. However, the number of sequences it can recognize is limited by its strict dependence on the PAM (5′-NGG-3′) motif (Zeng et al., 2020). In case non-canonical PAM motifs could be recognized, the number of sequences that can be edited would increase remarkably, for instance: 1.36 times in rice (Hu et al., 2016) and 1.62 in pepper (Li et al., 2020a). Therefore, several studies have focused on developing variants that improve PAM recognition sequence flexibility allowing broader screens on the target genome. Variants with different PAM preferences have been reported, such as VQR-Cas9 (NGA PAM), VRER-Cas9 (NGCG PAM), EQR-Cas9 (NGAG PAM; Kleinstiver et al., 2015), xCas9 (NG, GAA, and GTA PAM; Hu et al., 2018), SpCas9-NG (NG PAM; Nishimasu et al., 2018), SpG, and SpRY (Walton et al., 2020).

SpCas9-VQR, EQR, and VRER have been used to edit Arabidopsis (Yamamoto et al., 2019) and rice genomes (Hu et al., 2016, 2018), but their cleavage efficiency was lower in comparison with wild type SpCas9 (Kleinstiver et al., 2015; Hu et al., 2016, 2018; Yamamoto et al., 2019). On the other hand, SpCas9-NG allows a broader sequence recognition, identifying at least four types of atypical PAMs without showing a preference for the third nucleotide (NAC, NTG, NTT, and NCG; Hua et al., 2019; Ren et al., 2019a; Zhong et al., 2019) which increases the possibility of genetic editing in any given target of a plant genome. The most effective xCas9 variant is xCas9-3.7, showing high targeting fidelity, although a lower editing efficiency compared to SpCas9-NG (Hua et al., 2019; Zhong et al., 2019).

Interestingly, the development of variants partial or totally independent to the PAM sequence (SpG and SpRY) increases the possibility to edit a wider variety of genomic loci (Walton et al., 2020). Both variants, SpRY and SpCas9-NG, are so far the most efficient in the context of site-directed mutagenesis not only in plants (Ren et al., 2021; Xu et al., 2021) but also in single-celled organisms (Asano et al., 2021) Nevertheless, the relaxation that allows them to expand their compatibility with shorter PAM sequences can also lead to the recognition of a greater number of potential off-target sites (Endo et al., 2019). Comparison of the editing specificity of these variants with Cas9-WT demonstrated that SpCas9-NG has comparable editing activity (Hua et al., 2019; Zhong et al., 2019), while the broad flexibility of SpRY increases the editing of off-target sites in all plant genomes (Walton et al., 2020; Xu et al., 2021). Fortunately, it was shown that SpRY-HF1 can almost completely mitigate off-target site editing while increasing the fraction of total events edited at on-target sites in human cells (Walton et al., 2020).

### Improved Templates for Homologous Recombination Using Cas9

In eukaryotic cells, the two repair mechanisms, NHEJ and HR, compete with each other, and NHEJ is by far the preferred choice (Miyaoka et al., 2016). In the presence of a repair template containing a region with homology to the region flanking the DSB, HR can take place, replacing part of the gene of interest (Begemann et al., 2017; Oz et al., 2021; Wei et al., 2021). The frequency of HR in plants is very low (Butt et al., 2017; Miki et al., 2018; Que et al., 2019; Ali et al., 2020) and the delivery of the DNA template to different cell types in the right amount to stimulate recombination is not an easy task (Schindele et al., 2018; Huang and Puchta, 2019). HR can also be directed by the addition of an RNA template molecule. Li et al. (2016) used a pair of gRNA targeted to adjacent introns of the gene encoding 5-enolpyruvylshikimate-3-phosphate synthase (EPSPS). By adding a donor DNA template containing a few mutations the rice endogenous gene was replaced at a 2% frequency, producing glyphosate-resistant plants. With the use of one sgRNA targeting one intron the gene replacement frequency was 2.2%. Furthermore, the site-specific gene replacements were transmitted to the next generation.

Several methods for template delivery aiming to increase HR efficiency have been developed and the majority lies on the type and/or quantity of the molecule used as template. Other studies aimed to increase efficiency by co-localizing the donor template and the RNA-guided nuclease. Aird et al. (2018) demonstrated in mammal cells that a 30-fold increase in HDR can be obtained by covalently tethering a single-stranded oligonucleotide (ssODN) to the Cas9 complex using a HUH endonuclease, creating a stable complex between the ribonucleoprotein (RNP) and the ssODN without the need for its chemical alteration, changes in the sgRNA or additional proteins. Butt et al. (2017) verified that the template can be delivered as an RNA molecule fused with the sgRNA, producing one single RNA molecule that will set the specificity of the nuclease and serve as the HR template. However, this technique has some limitations, such as low efficiency, low versatility and the need for long homology arms. Lu et al. (2020) improved dsDNA template stability and consequently the insertion efficiency (compared to unmodified dsDNA and to ssDNA) by adding at the 5′ and 3′ ends of both strands two phosphorothioate linkages. This methodology was also efficient to insert short (<70 bp) and longer (526 and 2,049 bp) modified dsDNA donors. These authors also developed the tandem repeat-HDR strategy (TR-HDR) in which the desired insertion is flanked by a repeat sequence, increasing HR, providing a robust successful mean of base substitutions.

The integration efficiency can also be enhanced by the use of engineered DNA virus-based replicons to amplify the number of template copies available to stimulate HR. Several studies were successful, and insertion of desired DNA sequences were achieved in some species such as tomato (Čermák et al., 2015; Dahan-Meir et al., 2018), rice (Wang et al., 2017a), and hexaploid wheat (Gil-Humanes et al., 2017).

The microhomology-mediated end joining (MMEJ) pathway happens when the donor template has short homology arms (5–25 pb) to be used during the HR system (Nakade et al., 2014). Although this repair mechanism has low occurrence in G0/G1 phase compared to S and G2 phases of plant cell cycle (Truong et al., 2013) and is highly error-prone (García-Medel et al., 2019). Some studies suggest that MMEJ, especially in the hexaploid wheat, may be an effective HR strategy (Nakade et al., 2014; Wang et al., 2014; Arndell et al., 2019).

Recently, Schubert et al. (2021), from Integrated DNA Technologies (United States) evaluated a series of parameters affecting homologous recombination efficiency using single-stranded oligodeoxynucleotide (ssODN) as donor templates, probing 254 loci in Jurkat cells and another 239 loci in HAP1 cells. Among several parameters tested, it is worth noting the selection of the targeting strand is highly dependent on the loci and also on the cell type and that the position of the DSB site has a critical impact. Moreover, the introduction of blocking mutations, inhibiting the re-cleavage of the recombined product by Cas9, also had a positive effect, namely when targeting the PAM sequence. The company has a website^1^ that uses all their findings to create optimized sequences for homologous recombination. The evaluation of these rules to plant genome editing awaits further experimentation.

Cas9-coupled deaminases and prime editing techniques were developed as an alternative to overcome those problems and precisely and efficiently produce specific edits without both DSB and a repair template, as discussed below.

### Introducing Base Edition With Nuclease Fused Deaminases

**Figure 1:**
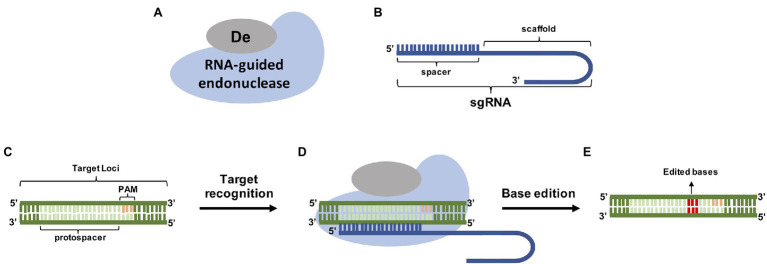
Genome editing using base editors **(A)** RNA-guided endonucleases and specific base Deaminases (De) are guided by a sgRNA **(B)** to its PAM containing target **(C)**. After target recognition **(D)** the deaminase precisely edited the bases inside the spacer site **(E)**.

Another variation of the CRISPR/Cas9 system is the base edition technique (Figure 1). Mutations are created due to the capacity of cytidine deaminases to convert C/G to A/T and adenine deaminases to convert A/T to C/G (Nishida et al., 2016; Schindele et al., 2018; Chen et al., 2019a). These editor proteins are fused to Cas9, Cas13, or Cas12a, that guide them to the desired locations in the genome. This approach allows the modification of only one nucleotide in a specific sequence. Using mammalian cells, it was shown that two mutations (Asp10Ala and His840Ala Cas9) can be introduced in the Cas9 protein, generating catalytic dead protein (dCas9) unable to produce a double-strand break, but still capable of being guided to a specific DNA target by a gRNA (Komor et al., 2016; Rees and Liu, 2018). The dCas9 protein is then fused with a deaminase allowing the substitution of the desired base. Amino acid substitution or stop codon generated by a single nucleotide mutation may lead to losing or changing the protein function. Komor et al. (2016) found that a Cas9 containing only the Asp10Ala mutation (Cas9 nickase, or nCas9) was able to introduce nicks at the target strand and performed better than dCas9. nCas9 also performed better than dCas9 in rice (Shimatani et al., 2017) and has been widely used in other plant species (Li et al., 2020c; Qin et al., 2020a).

More recently, several groups have reported the development of new deaminases able to perform transversion base changes, known as C-to-G base editors (CGBEs), by fusing rAPOBEC1 to either uracil DNA glycosylase (UNG; Kurt et al., 2021; Zhao et al., 2021) or XRCC1, a base excision repair protein (Chen et al., 2021). Besides their ability to perform new editions, a R33A mutation in the rAPOBEC1 deaminase and the use of UNG or rXRCC1, reduced off-targets of these new deaminases in the human genome (Chen et al., 2021; Kurt et al., 2021; Zhao et al., 2021). Interestingly, these authors also found that in the target sequences, these CGBE have a stronger bias towards the cytosine positioned at the sixth position. Sretenovic et al. (2021) produced versions of these three CGBE with codon optimization for plants, and their performance showed a dependence on the species. For example, in rice, monoallelic editing efficiency with a rXRCC1-based CGBE produced efficiencies up to 38%, while in poplar the best performance (6.25%) was obtained with the UNG-rAPOBEC1 (R33A)-based CGBE. Assays using rice and tomato protoplasts also showed C to T editions and indels as major byproducts. By contrast, in transgenic rice plants, no indels were observed in most CGBE constructs, indicating that these byproducts might be dependent on cell cycle and DNA repair mechanisms. It is worth noting that G to C edition had lower efficiency when compared to C to T conversion by the regular CBE system.

The use of nucleases that recognize different PAM can add versatility to genome editing. This is the case of Cas12a, which recognizes TTTV (V = A/G/C) as the PAM. However, Cas12a-based base editors (BEs) have lower editing efficiencies compared to SpCas9-based BE systems, probably because Cas12a has a looser binding to DNA targets when compared with SpCas9 (Bin Moon et al., 2018; Chen et al., 2020). The efficiency of base editing was also influenced by the context of the PAM sequence, indicating that the distinct gene context of the edition site should be carefully evaluated when using different combinations of nuclease:deaminase.

The addition of a protein from *Bacillus subtilis* bacteriophage PBS1, presenting uracil DNA glycosylase inhibitor (UGI), allowed a 3-fold enhancement in base edition in human cells (Komor et al., 2016). This is because this protein inhibits uracil DNA glycosylase (UDG), an enzyme involved in base-excision repair, reverting the edited U:G pair to the original C:G pair (Kunz et al., 2009). More recently, Qin et al. (2020b) produced an enhanced BE3 (eBE3), by adding three copies of UGI to the 3′ end of the BE3 sequence, which increased efficiency up to 2.8 times. Moreover, eBE3-edited plants had no indels which were observed in up to 25% of the edited plants using BE3 with a single UGI. The rates of undesired C>A and C>G conversion, and “clean edits” (only C>T substitutions) were 1.14–3.81 higher with eBE3 compared to BE3 in several gene targets. Interestingly, Nishida et al. (2016) also found evidence that UGI also helped to decrease indels at the edited site. It is worth noting that in their seminal work, Gaudelli et al. (2017) found that adenine BEs have lower rates of indels when compared to editions using Cas9. In plants, Jin et al. (2019) also found that adenine BEs have a low frequency of changes in off target sites, when compared to cytosine BEs.

The ability of the protein::RNA complex to be transported to the nucleus is a key step in genome editing. Koblan et al. (2018) found that a bipartite nuclear localization signal (bpNLS) positioned at the N and C terminus of the coding region of an APOBEC deaminase::Cas9::UGI fusion enhanced 1.3 fold the editing process in human cells. The same authors also found that the algorithms used by different authors and companies to optimize the coding regions also influenced the efficiency of the cytidine base editor, ranging from 20% to up to 60% of total sequencing reads with target C:G converted to T:A in human cells (Koblan et al., 2018). In the case of an adenine base editor, these authors found increases in editing efficiency from 1.3- to 7.9-fold when the optimization algorithms from two private companies were compared. Anyway, these enhancements are correlated with an improved nuclear localization of the editing protein fusion, as observed by Zafra et al. (2018), using the BE3 cytidine base editor in mice cells.

The influence of different NLS as well as the effect of multiple copies of NLS in base editing in rice was reported by Wang et al. (2020a). These authors found that in some genome sites, the efficiency of the different configurations of the base editor was similar. However, the efficiency was 3-fold higher in the case of the *OsSLR1* gene edited with a construct containing two different NLS compared to a construct containing one single copy. Li et al. (2018a), using a transient assay with a mutated version of the GFP gene in rice protoplasts, found no differences when constructs containing one or two NLS were used in vectors expressing an adenosine deaminase (ecTadA-ecTadA*) with nCas9 (D10A), while the insertion of a third copy of a NLS increased the efficiency by 25%. However, the increase in efficiency was less pronounced, around 10%, when several genes in the rice genome were assayed.

Changes in the sequence of the sgRNA can enhance A to G edition by more than two-fold in 13 target sites in both rice and wheat (Li et al., 2018a). Qin et al. (2020b) also observed editing frequencies of the enhanced gRNA (esgRNA) 1.9–2.1 fold higher than the usual gRNA in five genes from rice. These changes seem to increase not only the assembly of gRNA with the dCas9 protein, the gRNA stability, but also eliminate a putative Pol-III terminator (Chen et al., 2013). However, Wu et al. (2019) produced another esgRNA, introducing mutations in the native gRNA scaffold slightly different from those made by Qin et al. (2020), and observed only modest increases in BE efficiency. Although these studies tested the distinct esgRNA in different target genes, the data suggest that changes in the native gRNA need to be carefully tested.

## Prime Editing

**Figure 2:**
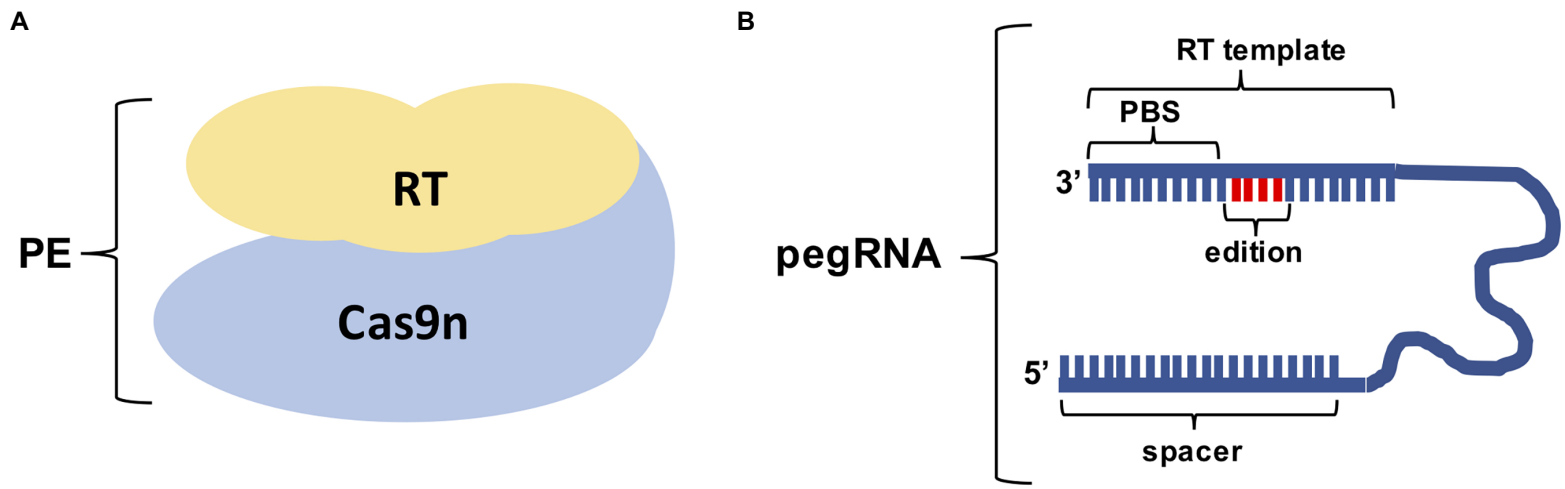
Components of the Prime editing technique. **(A)** Cas9 nickase (Cas9n) is fused to a Reverse Transcriptase (RT) to form the Prime editing protein complex. **(B)** The pegRNA contains the Prime Binding Site (PBS) which is used to prime the reverse transcriptase reaction, the template containing the desired edit and the spacer that will guide the Cas9n to the target.

**Figure 3:**
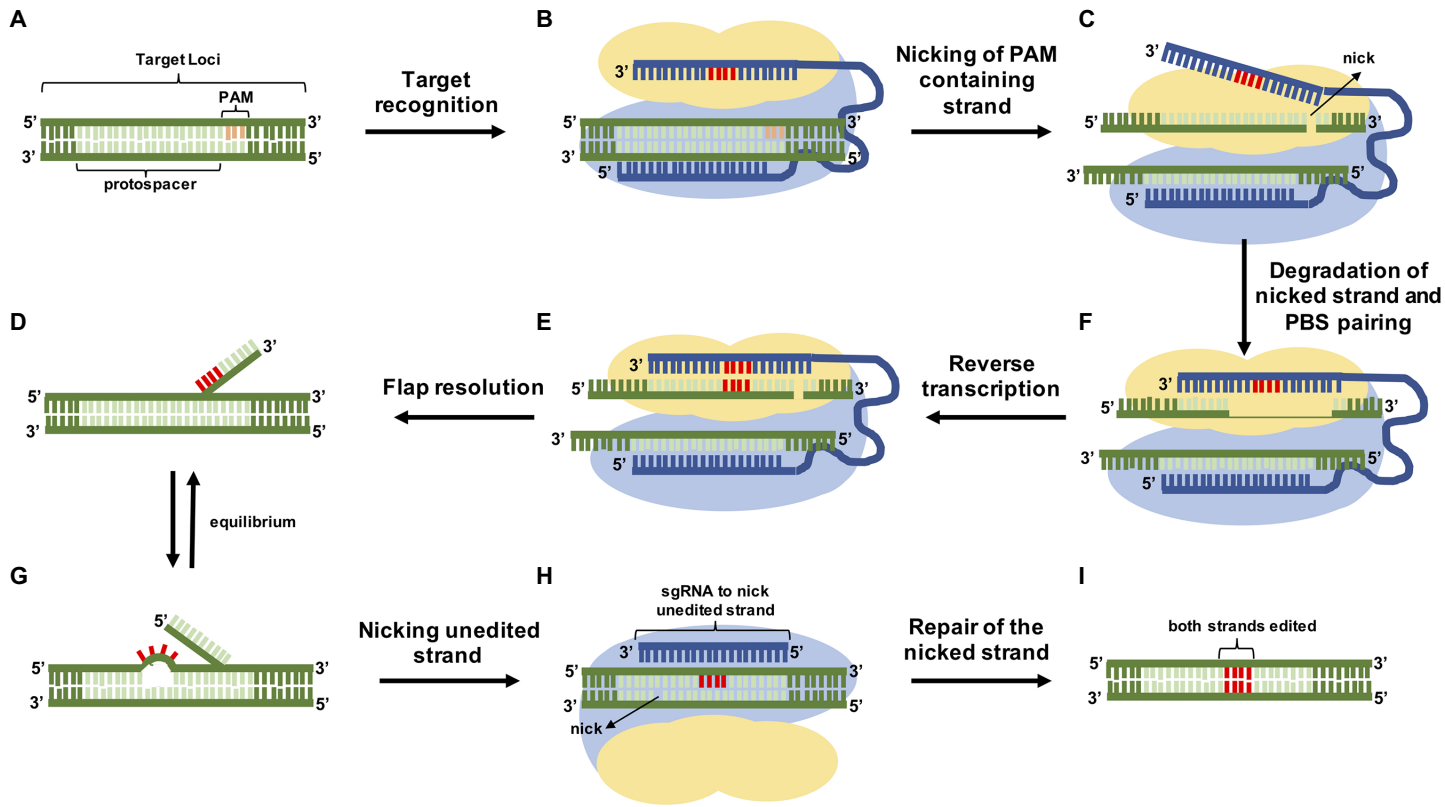
Overview of precise genome modification using prime editing. At the genomic site **(A)**, the nCas9 (H840A) is led to the target by the pegRNA **(B)** and breaks just the strand containing the PAM of the DNA duplex **(C)** exposing a 3′-hydroxyl group (3′ flap), that, together with the extension of the pegRNA PBS **(D)** is used to prime the RT reaction, inserting the edit **(E)**. The editing area contains two single-stranded flaps in equilibrium: the 3′ flap containing the edition **(F)** and the 5′ unedited flap **(G)**. The endogenous cellular endonucleases prefer 5′ flaps as a substrate digesting it and leaving the 3′ flap to be ligated. At the end of the process the nicked DNA strand is replaced by the newly synthesized strand that had the information copied from the pegRNA generating a heteroduplex. In this case, the repair mechanism is going to resolve the mismatch using one of the strands, so there is a 50% chance of the edition being repaired by the cell. To overcome this setback, the induction of a second nick on the unedited strand using a sgRNA **(H)** stimulates the repair by copying the information present on the edited strand **(I)**.

Prime editing was first described at the end of 2019 in human and yeast cells (Anzalone et al., 2019) and since then it has been used broadly in several plant species (Butt et al., 2020; Hua et al., 2020; Jiang et al., 2020; Li et al., 2020b; Lin et al., 2020; Xu et al., 2020a,b; Lu et al., 2021; Perroud et al., 2022). PE is based on a “search and replace” approach in which a nickase Cas9 (nCas9) is fused to a reverse transcriptase (RT), coupled to a prime editing guide RNA (pegRNA) that specifies both the target site and provides the template for the edition (Figure 2). The mechanism of PE action is described in Figure 3.

PE is divided into three main strategies: PE1 strategy 1 (PE1) uses a wild type Moloney murine leukemia virus (M-MLV) RT fused to a nCas9 and a pegRNA. PE strategy 2 (PE2) uses an engineered RT to increase the edition efficiency (mutations that affect thermostability, processivity, DNA–RNA substrate affinity and RNaseH activity) and therefore, exhibited a 1.6–5.1-fold improvement in introducing point mutations. PE strategy 3 (PE3) uses a second sgRNA to perform a nick on the non-edited strand to induce its replacement and increase the edition efficiency. PE strategy 3b (PE3b) uses a sgRNA that contains mismatches in order to be complementary to the edited strand, but not the original one, therefore, being active after the flap resolution. PE3b has 13-fold less indels compared to the PE3 in human cells (Anzalone et al., 2019).

The technique was successful to induced insertions (up to 44 nt), deletions (up to 80 nt), all types of transitions (C to T, G to A, A to G, and T to C), and transversions (C to A, C to G, G to C, G to T, A to C, A to T, T to A, and T to G) without inducing DBS. However, the authors describe that each prime editing experiment must be optimized because several factors can affect the efficiency, such as the source of RT enzyme, thermostability and binding capacity of the RT enzyme, length of the RT template, length of the PBS, position of the nicking sgRNA in the unmodified strand, secondary structure of the pegRNA, G/C content of the PBS, the target gene and location of the mutation relative to the PAM site (Anzalone et al., 2019).

In plants, these strategies have been tested in rice and the evidences show that PE3 does not increase the efficiency in relation to PE2 (Butt et al., 2020; Lin et al., 2020; Tang et al., 2020; Xu et al., 2020a). PE3b also does not increase the efficiency in relation to PE3 (Jiang et al., 2020; Lin et al., 2020; Xu et al., 2020a). For PE3 and PE3b a second nick is performed on the non-edit DNA strand to stimulate the repair using the new reverse transcribed strand as a template. Besides the different PE strategies, PBS, template length, nicking positions also have to be optimized for each target gene (Lin et al., 2020; Tang et al., 2020; Xu et al., 2020a,b; Lu et al., 2021). However, Lu et al. (2021) concluded that closer second nicks and simpler base changes affect the efficiency positively.

Lin et al. (2020) did an extensive evaluation of all types of base editions, deletions and insertion on several rice targets. They concluded that, although successful, each of them presented different efficiencies in different targets and that editing efficiencies decreased as the size of the intended deletions or insertions increased. Hua et al. (2020) showed in rice that only 9.1% of the regenerated plants were edited for a simple S627N change in the *OsALS* gene, while other targets (APO1, SLR1, OsSPL14, and APO2) aiming C42F change, 3-bp, 24-bp, and 24-bp insertion respectively, had no mutated lines. These data reinforce that the efficiency of even simple edits varies at different sites and longer insertions are harder to achieve. Xu et al. (2020a) using the *OsPDS* as a model analyzed different types of editions (insertion of 1, 2 or 3 nt; 28 bp deletion; A to T or A to C modification) and also observed a wide amplitude of edition frequencies (from 0 to 31.3%), with transversion being the most efficient edit.

### Optimizations of nCas9 and RT

The system can be modified by exchanging the nCas9-RT fusion and its expression. Hua et al. (2020) tested SaCas9, a multiple-turnover enzyme that releases DNA faster than SpCas9 (Yourik et al., 2019). SaCas9 was used to target the mutated version of the EGFP gene expressed in rice calli, but only 5.12% expressed GFP, much less than the 52.5–55.5% successfully edited using SpCas9. SaCas9 may require a different sequence and structure of sgRNA, and optimization of the pegRNA is required for this type of protein.

Xu et al. (2020b) fused a hygromycin phosphotransferase to the c-terminus of the nSpCas9-M-MLV region with a self-cleaving 2A peptide. Overall, most of the edits had their efficiency significantly increased (when compared to the nSPCas9-M-MLV alone) and it was especially efficient to induce multiple bases mutations.

Codon optimization and the use of alternative promoters are also strategies tested in order to increase PE efficiency. Tang et al. (2020) optimized both Cas9 and M-MLV codons for plants and added a nuclear localization signal and even though the efficiencies in rice protoplast were very low (0.1–1.55%), the optimization increased slightly the number of edited reads. Lu et al. (2021) also showed that codon optimization of the M-MLV RT increased by three times the editing efficiency in tomato, while the RPS5A promoter was also three times more effective than the usual 35S promoter.

Lin et al. (2020) evaluated the replacement of the engineered M-MLV RT by the cauliflower mosaic virus RT (RT-CaMV; Plant et al., 1985) or a retron-derived (RT-retron) from *Escherichia coli* BL21 (Lim and Maas, 1989) in an assay to convert the blue fluorescent protein (BFP) to GFP (Zong et al., 2017). Rice protoplasts were analyzed by flow cytometry and the M-MLV RT presented a 4.4% efficiency, RT-CaMV presented 3.7% and RT-retron 2.4%. M-MLV RT and RT-CaMV were also tested in an endogenous gene and showed significantly higher efficiency.

### pegRNA Optimizations

Some pegRNA design tools have been reported, such as Multicrispr (Bhagwat et al., 2020), PrimeDesign (Hsu et al., 2021), and PlantPegDesigner (Lin et al., 2021), but both of them are suitable for human/mouse genomes only. Despite that, pegRNAs can be optimized by changing their promoters, and therefore increasing their expression, changing the PBS or the template lengths. One study reported a high-throughput evaluation of PE2 activities in human cells using 54,836 pairs of pegRNAs to develop computational models to predict pegRNA efficiency and an accuracy by Spearman’s correlations was between 0.47 and 0.81. The author’s recommendations were: (1) use a 13-nt PBS and a 12-nt RT template; (2) use a high GC count in the PBS region if possible; (3) use a G at the last templated nucleotide when the RT template length is ≤12 nt; and (4) include PAM editing for human cells (Kim et al., 2021).

For plants, Lin et al. (2020) tested a variety of pegRNAs with differential PBS and RT template lengths and also nicking positions. The authors concluded that these factors strongly affect the editing frequencies in rice, and each target site presents optimal parameters. Jiang et al. (2020) hypothesized that the efficiency could be improved by enhancing the expression of pegRNAs. However, they verified that doubling the pegRNA expression cassettes in rice did not increase PE frequency.

The common conclusion of all studies that tested different PSB and RT length in plants, such as tomato (Lu et al., 2021), rice (Lin et al., 2020; Tang et al., 2020; Xu et al., 2020a,b), is that each target there has its optimal combination of PBS and template length, and for every study several tests must be performed to find the best strategy. Lin et al. (2021) described the in rice the optimal performance was achieved when the PBS had a melting temperature of 30°C and using two pegRNAs in trans coding for the same edits (called paired pegRNA) increased the edition efficiency from 2.9-fold to 17.4 fold and also launched a web application (PlantPegDesigner) for optimal pegRNA and paired pegRNA design.

A recent study (Liu et al., 2021) described the enhanced prime editing system (ePE) in which to avoid the circularization of the pegRNA due to complementarity between the PBS 3′ the spacer 5′ the 20-nt Csy4 recognition site was added to the 3′ end of the pegRNA. This site forms a hairpin therefore, avoiding pegRNA circularization and increased editing efficiency although the addition of Csy4 slightly increased indels frequency. This technique needs further optimization and there are no reports of its use in plants yet.

### Reducing Off-Targets in Prime Editing

Prime editing has a lower incidence of off-targets when compared to conventional CRISPR/Cas9 approach (Anzalone et al., 2020). The higher efficiency may be due to the three-step hybridization necessary for the editing. The first one is between the target DNA and the spacer present in the pegRNA, the second one between the PBS and the target DNA to start the RT priming and finally, the hybridization of the DNA flap. In the conventional CRISPR/Cas9 approach, only one hybridization is necessary, between the target DNA and the spacer in the sgRNA. The off-target rate in each experiment can also be influenced by the target DNA configuration in the chromosome, as heterochromatin or euchromatin (Kallimasioti-Pazi et al., 2018; Verkuijl and Rots, 2019). However, only one of all the studies in plants reported the evaluation of off-targets (Li et al., 2020b). Authors used the CRISPR-GE software^2^ and posterior PCR amplification and sequencing of 10 putative off targets of four genes targeted by prime editing, and they found no mutations, indicating a high specificity.

Kim et al. (2020a) developed a strategy named Nickase-based Digenome-seq (nDigenome-seq), based on next generation sequencing to recognize all off-targets produced by experiments using PE2 *in vitro* in human cells. They demonstrated that undesired DNA modifications were detected in 0.1–1.9% of the cases, which can be considered as very low. Despite the importance of this type of evaluation, only Li et al. (2020b) described that no off-targets were detected; however, several by-products such as chimeras and unexpected insertions were detected in rice plants. Kim et al. (2021) also found that PE specificity in human cells could be further improved by incorporating mutations from engineered Cas9 variants, particularly eSpCas9 and Sniper Cas9, into PE, and these strategies can also be used for plants.

One of the main pitfalls of PE technique is the relative high frequency of by-products such as incorporation of pegRNA scaffolds, incomplete editions due mistakes of the repair system and unexpected deletions and insertions. Reports indicate up to 37.2% frequency of by-products in maize (Jiang et al., 2020) and 2.23% in tomato (Xu et al., 2020b). In rice, by-products are also reported (Lin et al., 2020; Tang et al., 2020; Xu et al., 2020a; Li et al., 2020b). Lin et al. (2020), studying rice and wheat, tested several targets and pegRNAs for each one of them and concluded that different PBS length did not affect the frequency of by-products. Meanwhile, template length not only dramatically affected the proportion of by-products, but were also variable depending on the target locus. Interestingly, OsCDC48-T3 and OsEPSPS-T2 target sites were less edited, despite the high indel frequency generated by nCas9, indicating that prime editing activity is independent from Cas9 activity at some targets. Xu et al. (2020a) described that PE3 with second nick closer to the first original nicking site was more efficient in producing edited plants, but also produced a higher amount of plants with by-products.

Some studies were capable of regenerating plants of rice (Butt et al., 2020; Hua et al., 2020; Li et al., 2020b; Lin et al., 2020; Xu et al., 2020b), tomato (Lu et al., 2021), and maize (Jiang et al., 2020). However, despite all the efforts, these works have found that the production homozygous plants in the first generation has a very low efficiency. Most works reported heterozygous or chimeras which leads to the conclusion that this technique needs more improvement before being widely applied, namely in polyploid species.

## CRISPR/Cas12a System

**Figure 4:**
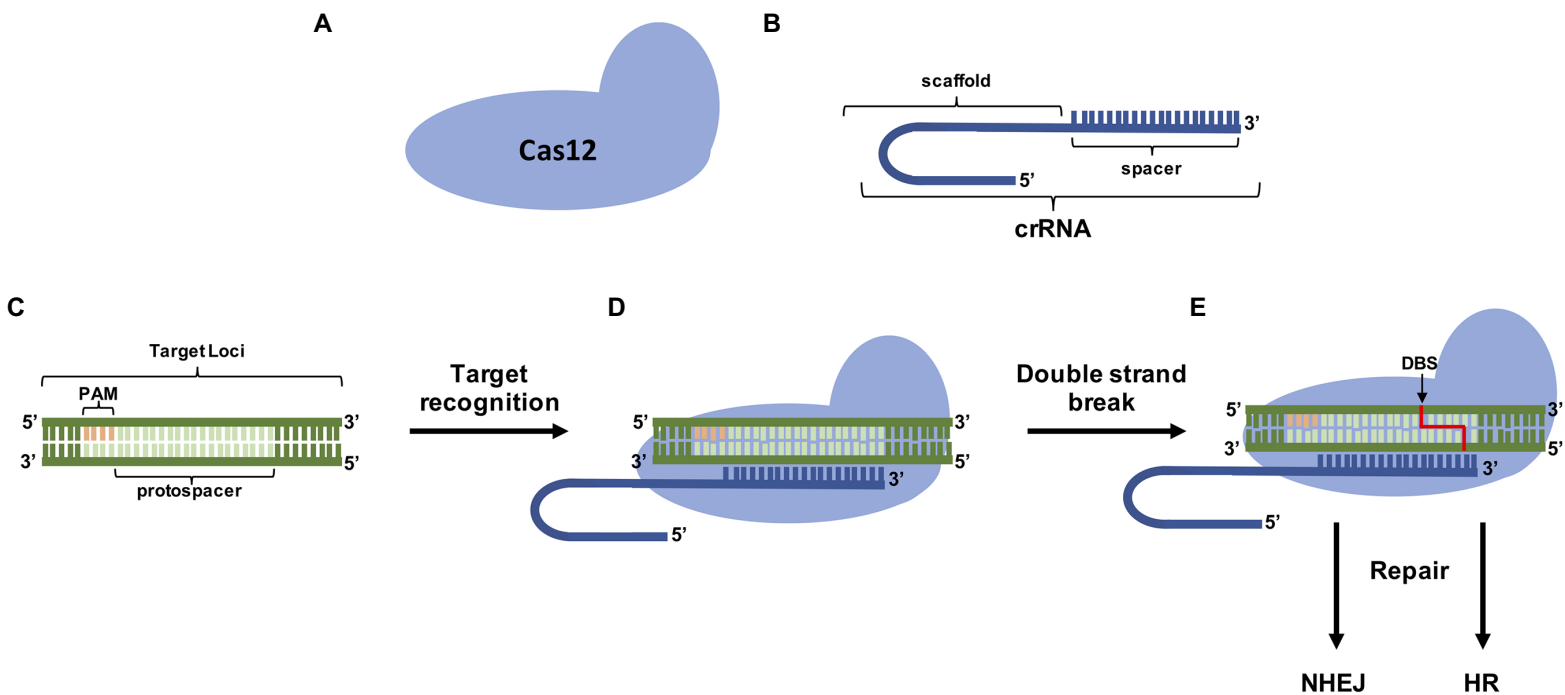
CRISPR/Cas12a genome editing components. **(A)** The Cas12a protein capable of provoking double-strand breaks (DSBs) in specific target sites with the help of **(B)** the CRISPR RNA (crRNA) that contains the sequence (spacer; **C**) complementary to the target 3′ from the Protospacer Adjacent Motif (PAM) site. **(D)** The ribonucleoprotein complex leads to specific DSB located generally cleaves DNA 18–23 nt distal of the PAM *via* 5 bp staggered double-stranded breaks. **(E)** Repair mechanisms (NHEJ and HR) fix DSBs.

The Cas12a endonuclease belongs to type V of class 2 effector proteins (Koonin et al., 2017) and has a size between ~1,200 and ~1,500 aa as a single subunit protein (Zetsche et al., 2015). Guided by a single mature crRNA of 42–44 nt length, Cas12a binds upstream of a typically thymidine-rich PAM TTTV (V = A, C, and G) and cleaves DNA 18–23 nt distal of the PAM *via* 5 bp staggered double-stranded breaks (Zetsche et al., 2015). Different from the Cas9 endonuclease which requires a tracrRNA and RNase III, Cas12a maturates the pre-crRNA by its intrinsic ribonuclease activities (Fonfara et al., 2016). The action mode of Cas12a is depicted at Figure 4.

The CRISPR/Cas12a system has been used mostly for mutagenesis through NHEJ repair in plants, but also HDR (Begemann et al., 2017; Li et al., 2019) and base editing have been demonstrated (Li et al., 2018b; Kleinstiver et al., 2020). With its great potential as a genome editing tool in plants, the CRISPR/Cas12a system has been improved to achieve higher editing efficiencies, more relaxed PAM requirements (Gao et al., 2017) and better efficiencies in multiplexed systems (Zhang et al., 2021).

### Assessment of “New” Cas12a Orthologs and Variants

In plants, mainly three Cas12a orthologs—FnCas12a (*Francisella novicida* Cas12a), LbCas12a (*Lachnospiraceae bacterium ND2006* Cas12a), and AsCas12a (*Acidaminococcus* sp. BV3L6)—have been used for genome editing (Zhong et al., 2018; Bandyopadhyay et al., 2020). Because of Cas12a’s restrictive PAM site requirement, TTTV, one major goal of researchers is to increase the PAM flexibility of Cas12a (Bandyopadhyay et al., 2020). While FnCas12a was initially shown to recognize TTV PAMs *in vitro* (Zetsche et al., 2015) and *in vivo* (Endo et al., 2016), Zhong et al. (2018) showed that in rice protoplasts FnCas12a mediated a high editing efficiency at all eight tested TTTV PAM sites (10–35%), while the efficiency at TTV PAM sites was relatively low (0–10%). A systematical comparison of all possible VTTV and VTTTV PAM sites while using the same protospacer sequences showed that ATTG/ATTTG, CTTC/CTTTC, and CTTG/CTTTG were equally edited by FnCas12a, while FnCas12a failed to edit only at GTTA or GTTC PAM sites.

The use of RR and RVR variants of AsCas12a and LbCas12a *in vitro* and in human cells were shown to overcome the strict requirement for TTTV PAM sites (Gao et al., 2017). In a similar fashion, LbCas12a-RR/FnCas12a-RR and LbCas12a-RVR/FnCas12a-RVR were tested in rice protoplasts at CCCC/TYCV PAMs (Y is C or T) and TATV PAMs, respectively (Zhong et al., 2018). The editing activity of LbCas12a-RR was tested at all tested CCCC and 10/11 TYCV PAM sites between 2 and 40% (11/13 higher than 10%) and outperformed FnCas12a-RR. The RVR variants were not able to mediate editing at TATC PAM sites and while both variants showed activity at two out of three TATG PAM sites, LbCas12a-RVR outperformed FnCas12a-RVR. In rice T0 plants LbCas12a-RR showed the highest and most robust editing frequency at two tested TTCC PAM sites (93.3 and 100%), while no or low editing activity of FnCas12a-RR at all tested PAM sites was observed.

Zhang et al. (2021) tested eight Cas12a orthologs in plants, which previously showed cleavage activity preferentially at TTV PAM sequences *in vitro* (Zetsche et al., 2020). From these, four orthologs [ErCas12a (MAD7, Inscripta, Inc.), Lb5Cas12a, BsCas12a, and Mb2Cas12a] showed high editing efficiencies at two different target sites in rice protoplasts (*OsDEP1-TTTC* and *OsEPFL9-TTTG*), which were comparable to those of LbCas12a (>30%). Also in transgenic rice lines, most of the tested Cas12a orthologs showed medium to high editing efficiencies at both target sites compared to LbCas12a.

Interestingly, Mb2Cas12a induced genome editing at 13 out of 18 tested VTTV PAM sequences with ~15% or higher efficiency. The editing efficiency of Mb2Cas12a at two more relaxed target sites (*OsROC5-GTTG* and *OsDEP1-GTTC*) was ~10% and Mb2Cas12a outperformed all other tested Cas12a orthologs. Introducing a RVR mutation into Mb2Cas12a (Mb2Cas12a-RVR) resulted in editing efficiencies from 15 to 35% at all TATV PAM sites which were higher than those from all other tested RVR variants (Zhang et al., 2021). The RVRR variant (Mb2Cas12a-RVRR) was active at TTTV, VTTV, TATV, TYCV, CCCV, and CTCV PAM sites and the genome-wide PAM analysis revealed that all targetable sites of Mb2Cas12a-RVRR cover 22.2% of the total rice genome (SpCas9 10.5%; Zhang et al., 2021). Recently, an engineered variant of LbCas12a (impLbCas12a) was shown to cleave TNTN PAMs with higher activity (Tóth et al., 2020) and could be a promising tool in plants.

### Overcoming Temperature Sensitivity of Cas12a

The above-mentioned temperature sensitivity of Cas12a nucleases is another drawback for their application in plants, because most plant transformation and cultivation processes are performed at lower temperatures (Bernabé-Orts et al., 2019; Lee et al., 2019; Malzahn et al., 2019). Malzahn et al. (2019) showed that AsCas12a, FnCas12a, and LbCas12a performed significantly better at temperatures ≥28°C compared to 22°C. Enhanced AsCas12a (enAsCas12a) showed two times higher activity at lower temperatures compared to AsCas12a in human cells (Kleinstiver et al., 2020), but was still outperformed by LbCas12a *in vitro* (Schindele and Puchta, 2020). Schindele and Puchta (2020) engineered, based on the findings from Kleinstiver et al. (2020), two LbCas12a variants (enLbCas12a and ttLbCas12a) and found that editing efficiencies of ttLbCas12a were 2- to 7-fold higher than those of LbCas12a at 22°C and were even higher than those of the other variants at 28°C.

Interestingly, Mb2Cas12a was less sensitive to lower temperatures (22°C) compared to other Cas12 orthologs and the activity could be further improved by introducing mutations equivalent to enAsCas12a (Kleinstiver et al., 2020; Zhang et al., 2021).

### Improving cRNA Design

Cas12a presents both endoribonuclease and endonuclease activity (Fonfara et al., 2016; Creutzburg et al., 2020). Consequently, it is not sufficient to predict Cas12a’s editing efficiency only based on the spacer-protospacer complementarity, but also on the efficiency of pre-crRNA maturation (Creutzburg et al., 2020). The maturation of the pre-crRNA is catalyzed by the ribonuclease domain of Cas12a after recognition of the hairpin structure formed by the repeat sequence at the 5′ end of the pre-crRNA (Fonfara et al., 2016; Creutzburg et al., 2020). The presence of surrounding nucleotides that base pair with nucleotides in the repeat sequence can result in alternative secondary structures which are not recognized by Cas12a (Creutzburg et al., 2020). While Creutzburg et al. (2020) provide valuable recommendations for crRNA design, it would be required to develop tools that predict efficient crRNAs also based on secondary structures.

While typically a crRNA of FnCas12a has a 24-nt guide sequence, Negishi et al. (2020) showed that the length of this guide sequence (18–30 nt) can positively influence the editing efficiency, while off-targets are not affected. It seems that there is an optimal length for each target sequence that must be tested individually. Tang et al. (2017) investigated the target specificity of LbCas12a by introducing double mismatch mutations into the protospacer and found that mismatches in the first 18 nt of the 23 nt of the protospacer abolished cleavage activity and mismatches at position 21–22 reduced cleavage activity by ~50%. These findings are in line with data from Zhang et al. (2021) that also confirm the high targeting specificity of several Cas12a orthologs.

### Increasing Multiplexing Efficiency in Cas12a Systems

Multiplex genome editing allows the introduction of two or more precise gene mutations in one plant generation and has the potential to significantly accelerate breeding processes (Abdelrahman et al., 2021). Several multiplex Cas12a systems in plants have been developed, but the efficiency of high biallelic editing frequencies in all targeted genes were very low (Wang et al., 2017b, 2018; Tang et al., 2019). An extensive comparison of different multiplexed systems indicated that the editing efficiency of all tested Pol II promoter-based systems were superior to Pol III promoter-based systems (Zhang et al., 2021). The most efficient combination was LbCas12a with a pZmUbi-HH-crRNA-HDV system, which resulted in 26/36 T_0_ lines carrying biallelic mutation in all four targeted genes. The same system, in combination with Mb2Cas12a, resulted in 8/11 T_0_ lines with biallelic mutations at all four sites. Mb2Cas12a-RVRR was able to edit seven targets with canonical and altered PAM sequences with editing efficiencies ranging from 5.1 to 31.1% in rice protoplasts. The power of this system was further demonstrated by simultaneously targeting 16 genomic sites across nine chromosomes in rice plants. While 20/21 T_0_ lines contained at least 13 edited sites of which at least seven were biallelic edits, 11/21 T_0_ lines had 15 edits and 1/21 T_0_ line showed editing in all 16 targeted genes. The proposed system allows editing of 14 sites simultaneously with biallelic mutations.

### Increasing HDR Efficiencies in Cas12a Systems

Cas12a is particularly suited for HDR as it induces a double-strand break 18 nt distal of the PAM and does not destroy the target sequence (Moreno-Mateos et al., 2017). Begemann et al. (2017) first demonstrated the capability of FnCas12a for an efficient targeted insertion in rice plants (8%), which performed better than LbCas12a under comparable conditions (up to 3%).

One strategy to improve the low HDR efficiency is to optimize the template delivery and Vu et al. (2020) utilized a geminiviral DNA replicon to express LbCas12a in tomato. The HDR efficiency mediated by LbCas12a expressed from the construct containing a geminiviral replicon (~4.5%) was significantly higher than the control and similar to the CRISPR/SpCas9-based construct (~3.6%). Further results showed that at higher temperatures (31°C) the efficiency can be significantly improved (9.8%). The use of a T-DNA system containing multiple replicons further increased the levels of donor template after the transformation step and resulted in higher HDR efficiencies up to 12.79%. In Arabidopsis, the variant ttLbCas12a outperformed LbCas12a at 22°C/28°C and ttLbCas12a showed a higher HDR efficiency at 22°C compared to LbCas12 at 28°C (Merker et al., 2020).

## CRISPR/Cas12b system

Cas12b (formerly C2c1) is a class 2 type V-B endonuclease and represents the third major CRISPR system that has been used for plant genome editing (Shmakov et al., 2015; Ming et al., 2020; Wang et al., 2020d; Wu et al., 2020). Like Cas12a, Cas12b recognizes T-rich PAM sequences, TTN, and generates staggered DSBs (7-nt 5′-overhangs) 23 bp upstream of the PAM on the non-target strand (Shmakov et al., 2015; Liu et al., 2017b). Unlike Cas12a, Cas12b is a dual-RNA-guided endonuclease which utilizes a cRNA and tracrRNA (or combined engineered sgRNA) for DNA recognition (Shmakov et al., 2015). The highest cleavage activity of Cas12b was achieved in the presence of Mn^2+^ and at temperatures between 37°C and 60°C (Shmakov et al., 2015; Yang et al., 2016; Liu et al., 2017b).

The first identified Cas12b orthologs, *Alicyclobacillus acidoterrestris* Cas12b (AacCas12b) and *Bacillus thermoamylovorans* Cas12b (BthCas12b) required high temperatures (48–50°C) for optimal activity *in vitro* (Shmakov et al., 2015). Wang et al. (2020d) used an AacCas12b-based system to edit the *GhCLA* gene in cotton, which as a thermophilic plant is able to survive higher temperatures. From three different temperatures (42°C, 45°C, and 48°C) and eight incubation times (ranging from 6 h to 15 days), the highest editing efficiency was achieved at 45°C after 4 days (Wang et al., 2020d).

The discovery of *Alicyclobacillus acidiphilus* Cas12b (AaCas12b; Teng et al., 2018) and later *Bacillus hisashii* Cas12b (BhCas12b; Strecker et al., 2019) which showed activity at lower temperatures, enabled the broader use of Cas12b for plant genome editing. In a comparative study, Ming et al. (2020) showed that AaCas12b has an editing efficiency of ~10% in rice protoplasts, while AacCas12b (~5%) and BthCas12b (very low) were less efficient. All three tested endonucleases generated 4–14 bp deletions located 12–24 bp distal to the PAM sites. Testing different PAM requirements for AaCas12b and AacCas12b revealed that both are capable of editing VTTV PAM sites with a preference for ATTV and GTTG sites. AaCas12b was further shown to be very sensitive to 2-pb mismatches in the protospacer and a protospacer length of ≤18 bp abolished the editing efficiency. 54.2% of the tested T_0_ lines were edited by AaCas12b and of these 46.2% of the T_0_ lines carried biallelic mutations.

Cas12b was further used in a multiplexed system with dual Pol II promoters and a hammerhead–sgRNA–hepatitis delta virus array to target three genes simultaneously (Ming et al., 2020). Compared to AacCas12b, AaCas12b performed better at two out of three target sites, but showed no activity at one site. At the two edited sites, 29.2 and 45.8% of the tested T_0_ line carried biallelic mutations.

## CRISPR/Cas12f System

Recently, two Class 2 miniature type V-F Cas nucleases, SpCas12f1 (497 aa) and AsCas12f1 (422 aa), have been used for genome editing in maize (Bigelyte et al., 2021). With their compact size compared to Cas9 and Cas12 endonucleases, Cas12f nucleases could be a good choice when using viral-based delivery systems. Furthermore, their dependence on higher temperatures for optimal activity (45–55°C) could help to better control off-target effects in some plant species (Bigelyte et al., 2021).

## Final Remarks

There is an urgent need to improve crop sustainability and the use of gene editing technologies will not only to increase our ability to modify traits already present in the crop, but also introduce new traits that so far have only been achieved with regular transgenesis, such as tolerance to herbicides (Dong et al., 2021) and insects (Tyagi et al., 2020). The use of deep learning technologies certainly will help our ability to engineer new proteins, with not only higher specificity, but also higher efficiency. In this sense, there is a plethora of organisms that have CRISPR-like system, including extremophyles, that remain to be explored (Salwan et al., 2020). In Table 1, we have compiled components that can be manipulated in order to increase the editing efficiency of CRISPR-based methods. It is worth mentioning that high throughput methods to screen new versions of all the editing components will improve our ability to discover new improvements in gRNA and protein structures, new editing features, among others.

**Table 1:** Overview of tools and techniques to increase genome editing efficiency.

Component	Strategies	Impact	Future developments
Guide RNA/pegRNA	– New computational algorithms allow the design of gRNA sequences with high affinity and specificity to the target sites, as well as higher affinity with the nuclease – Larger gRNAs (pegRNA) provide an HR template when combined with nuclease/reverse transcriptases	– Reduced binding to non-target sites – Enhanced binding to target sites – Higher nuclease activity	– Improved algorithms to decipher the best gRNA sequence – Improve prediction for best optimal combination of PBS and template length
Nucleases	– Protein engineering to produce evolved nucleases – Association with proteins or domains with new features – Discovery of new nucleases	– Higher DNA cutting activity – Increased affinity to gRNA – Broader target selection, allowing to edit the entire genome (PAM-less nucleases) – New functions by fusion with other proteins, such as deaminases and reverse transcriptase – Improved nuclear localization – Higher activity at temperatures used in plant cell tissue culture	– Computational tools to create new protein structures – Identification of proteins from a broader range of organisms, with new abilities – Increase the editing power in order to decrease chimera or heterologous plants
Deaminase	– Protein engineering – Fusions with new proteins or protein domains	– New editing capacities, such as transversions – Higher predictability of the edited base	– Discovery of new deaminases or other protein modules that can improve the editing process
HR templates	– Increased template availability by using synthetic DNA more resistant to nucleases, higher copy number using virus replicons, and co-localization of the HR template to the RNP complex by fusions with the gRNA	– Higher probability of HR, therefore, increased desired editing rate	– Better understanding of key cellular components involved in HR enhancement and also in HR inhibition – New strategies to increase HR stability and proximity to the editing site
Computational tools	– Identification of new nuclease variants with increased specificity and efficiency – Increased accuracy of both target and off-target predictions		– New experiments designed to gather data to feed the algorithms – New algorithms and publicly available servers – Decipher the role of epigenomics and chromatin structure in the gRNA binding and nuclease activity
Plant tissue culture	– Protocols that allow the selection of edited cells without transgenes, due to mutations in genes encoding herbicide tolerance	– GMO-free edited plants	– Multiplex editions, targeting the herbicide tolerance gene and other genes related to other traits
Proteins components in general	– Codon optimization – Promoters and terminators with higher activity	– Enhanced levels of the proteins involved in gene editing	– Identification of new promoters and terminators with higher activity in the cell types and initial stages of the transformation

Genome editing technologies are a new addition to the toolbox of genetic engineering that so far has relied extensively in the addition of DNA sequences from other organisms. In spite of all the scientific evidence indicating genetically modified organisms (GMOs) are safe for human consumption, skepticism still remains among consumers, with 46.7% having a negative view on GM food in China (Cui and Shoemaker, 2018), 57% in the United States (Pew Research Center, 2015), and 34.7% in Canada (Charlebois et al., 2019). Although increased knowledge from qualified sources are known to move this trend towards a positive view on GMOs (Wunderlich and Gatto, 2015; Hunt and Wald, 2021), there is evidence that precision genome editing, without the incorporation of recombinant DNA in plant genomes will have a higher acceptance than GMOs because no exogenous DNA would be introduced (Muringai et al., 2020).

Additionally, after all components of the gene-editing system have been integrated into the host genome, chances of off-target indels increase with time (Lawrenson et al., 2015). The capability of producing transgenic plants without the integration of foreign DNA would alleviate the regulatory concerns in order to commercialize new varieties (Jones, 2015).

The delivery of reagents for genome editing without incorporating DNA into the genome results in mutations identical to naturally occurring events. This approach, also called DNA-free genome editing, has been reported in protoplast from several plant species with a mutagenesis frequency of up to 46% in Arabidopsis, tobacco, rice, and lettuce plants (Woo et al., 2015; Kim et al., 2017). However, regeneration of plants from transformed protoplasts, especially monocots, can be challenging. To overcome this, Zhang et al. (2016) used biolistics to introduce Cas9 mRNA and gRNA into immature wheat embryos that were allowed to regenerate plants without selection. This strategy resulted in genome editing at a ratio of 1.1 plants per 100 bombarded embryos. In addition, transformed plants were transgene-free, since no DNA was used in the process.

The use of DNA-free CRISPR/Cas techniques in crop plants is particularly interesting due to the regulatory issues involving the release of commercial products from genetically modified organisms. Recently, the USDA-APHIS confirmed that plants with phenotypes developed through genome editing without foreign DNA inserted in the genome will not require regulatory approval for commercial production in the United States (Waltz, 2016). Products to be commercialized will be evaluated on a case-by-case basis, but certainly will result in a remarkable reduction of regulatory costs for cultivar development. In Brazil, genome editing technologies will not be regulated as GMOs, on a case-by-case analysis (CTNBio, 2018). On the contrary, in Europe, the decision on regulating precision breeding technologies as GMOs has created concerns on the continent’s economy (Hjort et al., 2021). On a global scale, local authorities are still discussing regulatory frameworks for genome-edited plants in other countries. In the meantime, delivery methods have been developed that introduce targeted mutations without any transgenic footprint of the genome editing tools (Liang et al., 2017). This transformative technology will allow a paradigm shift in improvement and streamline regulatory approval of genetically modified crops.

## Author Contributions

LM, MR, MS, RT, JS, KB, and MM: wrote and reviewed the paper. All authors contributed to the article and approved the submitted version.

## Funding

This work was supported by the Competitive Seed Grant Research Initiative (Grant No. 52900129910) from the College of Agricultural and Life Sciences at the University of Florida (KB) and Fundação de Amparo à Pesquisa do Estado de São Paulo and BG E&P Brasil, Research Centre for Gas Innovation (Grant No. 2020/15230-5; MM).

## Conflict of Interest

The authors declare that the research was conducted in the absence of any commercial or financial relationships that could be construed as a potential conflict of interest.

## Publisher’s Note

All claims expressed in this article are solely those of the authors and do not necessarily represent those of their affiliated organizations, or those of the publisher, the editors and the reviewers. Any product that may be evaluated in this article, or claim that may be made by its manufacturer, is not guaranteed or endorsed by the publisher.

## Footnotes

^1^ https://www.idtdna.com/HDR

^2^ http://skl.scau.edu.cn/

## References

[ref1] Abdelrahman M. Wei Z. Rohila J. S. Zhao K. (2021). Multiplex genome-editing technologies for revolutionizing plant biology and crop improvement. Front. Plant Sci. 12:721203. doi: 10.3389/fpls.2021.721203, PMID: 34691102 PMC8526792

[ref2] Aird E. J. Lovendahl K. N. St. Martin A. Harris R. S. Gordon W. R. (2018). Increasing Cas9-mediated homology-directed repair efficiency through covalent tethering of DNA repair template. Commun. Biol. 1:54. doi: 10.1038/s42003-018-0054-2, PMID: 30271937 PMC6123678

[ref3] Ali Z. Shami A. Sedeek K. Kamel R. Alhabsi A. Tehseen M.. (2020). Fusion of the Cas9 endonuclease and the VirD2 relaxase facilitates homology-directed repair for precise genome engineering in rice. Commun. Biol. 3:44. doi: 10.1038/s42003-020-0768-9, PMID: 31974493 PMC6978410

[ref4] Alley E. C. Khimulya G. Biswas S. AlQuraishi M. Church G. M. (2019). Unified rational protein engineering with sequence-based deep representation learning. Nat. Methods 16, 1315–1322. doi: 10.1038/s41592-019-0598-1, PMID: 31636460 PMC7067682

[ref5] Anzalone A. V. Koblan L. W. Liu D. R. (2020). Genome editing with CRISPR–Cas nucleases, base editors, transposases and prime editors. Nat. Biotechnol. 38, 824–844. doi: 10.1038/s41587-020-0561-9, PMID: 32572269

[ref6] Anzalone A. V. Randolph P. B. Davis J. R. Sousa A. A. Koblan L. W. Levy J. M.. (2019). Search-and-replace genome editing without double-strand breaks or donor DNA. Nature 576, 149–157. doi: 10.1038/s41586-019-1711-4, PMID: 31634902 PMC6907074

[ref7] Arndell T. Sharma N. Langridge P. Baumann U. Watson-Haigh N. S. Whitford R. (2019). gRNA validation for wheat genome editing with the CRISPR-Cas9 system. BMC Biotechnol. 19:71. doi: 10.1186/s12896-019-0565-z, PMID: 31684940 PMC6829922

[ref8] Asano Y. Yamashita K. Hasegawa A. Ogasawara T. Iriki H. Muramoto T. (2021). Knock-in and precise nucleotide substitution using near-PAMless engineered Cas9 variants in Dictyostelium discoideum. Sci. Rep. 11:11163. doi: 10.1038/s41598-021-89546-0, PMID: 34045481 PMC8159936

[ref9] Banakar R. Schubert M. Collingwood M. Vakulskas C. Eggenberger A. L. Wang K. (2020). Comparison of CRISPR-Cas9/Cas12a Ribonucleoprotein complexes for genome editing efficiency in the Rice Phytoene Desaturase (OsPDS) gene. Rice 13:4. doi: 10.1186/s12284-019-0365-z, PMID: 31965382 PMC6973557

[ref10] Bandyopadhyay A. Kancharla N. Javalkote V. S. Dasgupta S. Brutnell T. P. (2020). CRISPR-Cas12a (Cpf1): a versatile tool in the plant genome editing tool box for agricultural advancement. Front. Plant Sci. 11:584151. doi: 10.3389/fpls.2020.584151, PMID: 33214794 PMC7668199

[ref11] Begemann M. B. Gray B. N. January E. Gordon G. C. He Y. Liu H.. (2017). Precise insertion and guided editing of higher plant genomes using Cpf1 CRISPR nucleases. Sci. Rep. 7:11606. doi: 10.1038/s41598-017-11760-6, PMID: 28912524 PMC5599503

[ref12] Belhaj K. Chaparro-Garcia A. Kamoun S. Nekrasov V. (2013). Plant genome editing made easy: targeted mutagenesis in model and crop plants using the CRISPR/Cas system. Plant Methods 9:39. doi: 10.1186/1746-4811-9-39, PMID: 24112467 PMC3852272

[ref13] Bernabé-Orts J. M. Casas-Rodrigo I. Minguet E. G. Landolfi V. Garcia-Carpintero V. Gianoglio S.. (2019). Assessment of Cas12a-mediated gene editing efficiency in plants. Plant Biotechnol. J. 17, 1971–1984. doi: 10.1111/pbi.13113, PMID: 30950179 PMC6737022

[ref14] Bhagwat A. M. Graumann J. Wiegandt R. Bentsen M. Welker J. Kuenne C.. (2020). Multicrispr: gRNA design for prime editing and parallel targeting of thousands of targets. Life Sci. Alliance 3:e202000757. doi: 10.26508/lsa.202000757, PMID: 32907859 PMC7494814

[ref15] Bigelyte G. Young J. K. Karvelis T. Budre K. Zedaveinyte R. Djukanovic V.. (2021). Miniature type V-F CRISPR-Cas nucleases enable targeted DNA modification in cells. Nat. Commun. 12:6191. doi: 10.1038/s41467-021-26469-4, PMID: 34702830 PMC8548392

[ref16] Bin Moon S. Lee J. M. Kang J. G. Lee N.-E. Ha D.-I. Kim D. Y.. (2018). Highly efficient genome editing by CRISPR-Cpf1 using CRISPR RNA with a uridinylate-rich 3′-overhang. Nat. Commun. 9:3651. doi: 10.1038/s41467-018-06129-w, PMID: 30194297 PMC6128929

[ref17] Biswas S. Khimulya G. Alley E. C. Esvelt K. M. Church G. M. (2021). Low-N protein engineering with data-efficient deep learning. Nat. Methods 18, 389–396. doi: 10.1038/s41592-021-01100-y, PMID: 33828272

[ref18] Boyle E. A. Becker W. R. Bai H. B. Chen J. S. Doudna J. A. Greenleaf W. J. (2021). Quantification of Cas9 binding and cleavage across diverse guide sequences maps landscapes of target engagement. Sci. Adv. 7:eabe5496. doi: 10.1126/sciadv.abe5496, PMID: 33608277 PMC7895440

[ref19] Butt H. Eid A. Ali Z. Atia M. A. M. Mokhtar M. M. Hassan N.. (2017). Efficient CRISPR/Cas9-mediated genome editing using a chimeric single-guide RNA molecule. Front. Plant Sci. 8:1441. doi: 10.3389/fpls.2017.01441, PMID: 28883826 PMC5573723

[ref20] Butt H. Rao G. S. Sedeek K. Aman R. Kamel R. Mahfouz M. (2020). Engineering herbicide resistance via prime editing in rice. Plant Biotechnol. J. 18, 2370–2372. doi: 10.1111/pbi.13399, PMID: 32415890 PMC7680537

[ref21] Čermák T. Baltes N. J. Čegan R. Zhang Y. Voytas D. F. (2015). High-frequency, precise modification of the tomato genome. Genome Biol. 16:232. doi: 10.1186/s13059-015-0796-9, PMID: 26541286 PMC4635538

[ref22] Charlebois S. Somogyi S. Music J. Cunningham C. (2019). Biotechnology in food: Canadian attitudes towards genetic engineering in both plant- and animal-based foods. Br. Food J. 121, 3181–3192. doi: 10.1108/BFJ-07-2018-0471

[ref001] Chen B. Gilbert L. A. Cimini B. A. Schnitzbauer J. Zhang W. Li G. W.. (2013). Dynamic imaging of genomic loci in living human cells by an optimized CRISPR/Cas system. Cell 55, 1479–1491. doi: 10.1016/j.cell.2013.12.001, PMID: 24360272 PMC3918502

[ref23] Chen W. McKenna A. Schreiber J. Haeussler M. Yin Y. Agarwal V.. (2019b). Massively parallel profiling and predictive modeling of the outcomes of CRISPR/Cas9-mediated double-strand break repair. Nucleic Acids Res. 47, 7989–8003. doi: 10.1093/nar/gkz487, PMID: 31165867 PMC6735782

[ref002] Chen S. Jia Y. Liu Z. (2020). Robustly improved base editing efficiency of Cpf1 base editor using optimized cytidine deaminases. Cell 6:62. doi: 10.1038/s41421-020-00195-5, PMID: 33014423 PMC7490413

[ref24] Chen L. Park J. E. Paa P. Rajakumar P. D. Prekop H.-T. Chew Y. T.. (2021). Programmable C:G to G:C genome editing with CRISPR-Cas9-directed base excision repair proteins. Nat. Commun. 12:1384. doi: 10.1038/s41467-021-21559-9, PMID: 33654077 PMC7925527

[ref25] Chen K. Wang Y. Zhang R. Zhang H. Gao C. (2019a). CRISPR/Cas genome editing and precision plant breeding in agriculture. Annu. Rev. Plant Biol. 70, 667–697. doi: 10.1146/annurev-arplant-050718-100049, PMID: 30835493

[ref26] Chuai G. Ma H. Yan J. Chen M. Hong N. Xue D.. (2018). DeepCRISPR: optimized CRISPR guide RNA design by deep learning. Genome Biol. 19:80. doi: 10.1186/s13059-018-1459-4, PMID: 29945655 PMC6020378

[ref27] Creutzburg S. C. A. Wu W. Y. Mohanraju P. Swartjes T. Alkan F. Gorodkin J.. (2020). Good guide, bad guide: spacer sequence-dependent cleavage efficiency of Cas12a. Nucleic Acids Res. 48, 3228–3243. doi: 10.1093/nar/gkz1240, PMID: 31989168 PMC7102956

[ref28] CTNBio (2018). Resolução Normativa No 16, de 15 de janeiro de 2018 - Resoluções Normativas - Comissão Técnica Nacional de Biossegurança$ &ndash CTNBio. Available at: http://ctnbio.mctic.gov.br/en/resolucoes-normativas/-/asset_publisher/OgW431Rs9dQ6/content/resolucao-normativa-n%C2%BA-16-de-15-de-janeiro-de-2018;jsessionid=14341B56AB1AC78EF2817941CF29950F.columba (Accessed December 7, 2021).

[ref29] Cui K. Shoemaker S. P. (2018). Public perception of genetically-modified (GM) food: a Nationwide Chinese consumer study. NPJ Sci. Food 2:10. doi: 10.1038/s41538-018-0018-4, PMID: 31304260 PMC6550219

[ref30] Curtin S. J. Voytas D. F. Stupar R. M. (2012). Genome engineering of crops with designer nucleases. Plant Genome 5, 42–50. doi: 10.3835/plantgenome2012.06.0008, PMID: 28806883

[ref31] Dahan-Meir T. Filler-Hayut S. Melamed-Bessudo C. Bocobza S. Czosnek H. Aharoni A.. (2018). Efficient in planta gene targeting in tomato using geminiviral replicons and the CRISPR/Cas9 system. Plant J. 95, 5–16. doi: 10.1111/tpj.13932, PMID: 29668111

[ref32] Doench J. G. Hartenian E. Graham D. B. Tothova Z. Hegde M. Smith I.. (2014). Rational design of highly active sgRNAs for CRISPR-Cas9–mediated gene inactivation. Nat. Biotechnol. 32, 1262–1267. doi: 10.1038/nbt.3026, PMID: 25184501 PMC4262738

[ref33] Dong H. Huang Y. Wang K. (2021). The development of herbicide resistance crop plants using CRISPR/Cas9-mediated gene editing. Genes 12:912. doi: 10.3390/genes12060912, PMID: 34204760 PMC8231513

[ref34] Endo A. Masafumi M. Kaya H. Toki S. (2016). Efficient targeted mutagenesis of rice and tobacco genomes using Cpf1 from Francisella novicida. Sci. Rep. 6:38169. doi: 10.1038/srep38169, PMID: 27905529 PMC5131344

[ref35] Endo M. Mikami M. Endo A. Kaya H. Itoh T. Nishimasu H.. (2019). Genome editing in plants by engineered CRISPR–Cas9 recognizing NG PAM. Nat. Plants 5, 14–17. doi: 10.1038/s41477-018-0321-8, PMID: 30531939

[ref36] Feng X. Peng C. Chen Y. Liu X. Feng X. He Y. (2017). Discrimination of CRISPR/Cas9-induced mutants of rice seeds using near-infrared hyperspectral imaging. Sci. Rep. 7:15934. doi: 10.1038/s41598-017-16254-z, PMID: 29162881 PMC5698449

[ref37] Fonfara I. Richter H. Bratovič M. Le Rhun A. Charpentier E. (2016). The CRISPR-associated DNA-cleaving enzyme Cpf1 also processes precursor CRISPR RNA. Nature 532, 517–521. doi: 10.1038/nature17945, PMID: 27096362

[ref38] Gao L. Cox D. B. T. Yan W. X. Manteiga J. C. Schneider M. W. Yamano T.. (2017). Engineered Cpf1 variants with altered PAM specificities. Nat. Biotechnol. 35, 789–792. doi: 10.1038/nbt.3900, PMID: 28581492 PMC5548640

[ref39] Gao W. Mahajan S. P. Sulam J. Gray J. J. (2020). Deep learning in protein structural Modeling and design. Patterns 1:100142. doi: 10.1016/j.patter.2020.100142, PMID: 33336200 PMC7733882

[ref40] García-Medel P. L. Baruch-Torres N. Peralta-Castro A. Trasviña-Arenas C. H. Torres-Larios A. Brieba L. G. (2019). Plant organellar DNA polymerases repair double-stranded breaks by microhomology-mediated end-joining. Nucleic Acids Res. 47, 3028–3044. doi: 10.1093/nar/gkz039, PMID: 30698803 PMC6451138

[ref41] Gaudelli N. M. Komor A. C. Rees H. A. Packer M. S. Badran A. H. Bryson D. I.. (2017). Programmable base editing of A•T to G•C in genomic DNA without DNA cleavage. Nature 551, 464–471. doi: 10.1038/nature24644, PMID: 29160308 PMC5726555

[ref42] Gil-Humanes J. Wang Y. Liang Z. Shan Q. Ozuna C. V. Sánchez-León S.. (2017). High-efficiency gene targeting in hexaploid wheat using DNA replicons and CRISPR/Cas9. Plant J. 89, 1251–1262. doi: 10.1111/tpj.13446, PMID: 27943461 PMC8439346

[ref43] Griggs D. Stafford-Smith M. Gaffney O. Rockström J. Öhman M. C. Shyamsundar P.. (2013). Sustainable development goals for people and planet. Nature 495, 305–307. doi: 10.1038/495305a, PMID: 23518546

[ref44] Grützner R. Martin P. Horn C. Mortensen S. Cram E. J. Lee-Parsons C. W. T.. (2021). High-efficiency genome editing in plants mediated by a Cas9 gene containing multiple introns. Plant Commun. 2:100135. doi: 10.1016/j.xplc.2020.100135, PMID: 33898975 PMC8060730

[ref45] Hajiahmadi Z. Movahedi A. Wei H. Li D. Orooji Y. Ruan H.. (2019). Strategies to increase on-target and reduce off-target effects of the CRISPR/Cas9 system in plants. IJMS 20:3719. doi: 10.3390/ijms20153719, PMID: 31366028 PMC6696359

[ref46] Hjort C. Cole J. Frébort I. (2021). European genome editing regulations: threats to the European bioeconomy and unfit for purpose. EFB Bioecon. J. 1:100001. doi: 10.1016/j.bioeco.2021.100001

[ref47] Howells R. M. Craze M. Bowden S. Wallington E. J. (2018). Efficient generation of stable, heritable gene edits in wheat using CRISPR/Cas9. BMC Plant Biol. 18:215. doi: 10.1186/s12870-018-1433-z, PMID: 30285624 PMC6171145

[ref48] Hsu J. Y. Grünewald J. Szalay R. Shih J. Anzalone A. V. Lam K. C.. (2021). PrimeDesign software for rapid and simplified design of prime editing guide RNAs. Nat. Commun. 12:1034. doi: 10.1038/s41467-021-21337-7, PMID: 33589617 PMC7884779

[ref49] Hsu P. D. Lander E. S. Zhang F. (2014). Development and applications of CRISPR-Cas9 for genome engineering. Cell 157, 1262–1278. doi: 10.1016/j.cell.2014.05.010, PMID: 24906146 PMC4343198

[ref50] Hu J. H. Miller S. M. Geurts M. H. Tang W. Chen L. Sun N.. (2018). Evolved Cas9 variants with broad PAM compatibility and high DNA specificity. Nature 556, 57–63. doi: 10.1038/nature26155, PMID: 29512652 PMC5951633

[ref51] Hu X. Wang C. Fu Y. Liu Q. Jiao X. Wang K. (2016). Expanding the range of CRISPR/Cas9 genome editing in rice. Mol. Plant 9, 943–945. doi: 10.1016/j.molp.2016.03.003, PMID: 26995294

[ref52] Hua K. Jiang Y. Tao X. Zhu J. (2020). Precision genome engineering in rice using prime editing system. Plant Biotechnol. J. 18, 2167–2169. doi: 10.1111/pbi.13395, PMID: 32372479 PMC7589318

[ref53] Hua K. Tao X. Han P. Wang R. Zhu J.-K. (2019). Genome engineering in rice using Cas9 variants that recognize NG PAM sequences. Mol. Plant 12, 1003–1014. doi: 10.1016/j.molp.2019.03.009, PMID: 30928636

[ref54] Huang T.-K. Puchta H. (2019). CRISPR/Cas-mediated gene targeting in plants: finally a turn for the better for homologous recombination. Plant Cell Rep. 38, 443–453. doi: 10.1007/s00299-019-02379-0, PMID: 30673818

[ref55] Hunt K. P. Wald D. M. (2021). Full article: the role of scientific source credibility and goodwill in public skepticism toward GM foods. Available at: https://www.tandfonline.com/doi/full/10.1080/17524032.2020.1725086 (Accessed December 7, 2021).

[ref56] Jiang Y.-Y. Chai Y.-P. Lu M.-H. Han X.-L. Lin Q. Zhang Y.. (2020). Prime editing efficiently generates W542L and S621I double mutations in two ALS genes in maize. Genome Biol. 21:257. doi: 10.1186/s13059-020-02170-5, PMID: 33023639 PMC7541250

[ref57] Jin S. Zong Y. Gao Q. Zhu Z. Wang Y. Qin P.. (2019). Cytosine, but not adenine, base editors induce genome-wide off-target mutations in rice. Science 364, 292–295. doi: 10.1126/science.aaw7166, PMID: 30819931

[ref003] Jones H. (2015). Regulatory uncertainty over genome editing. Nat. Plants. 1:14011., PMID: 27246057 10.1038/nplants.2014.11

[ref58] Jung C. Till B. (2021). Mutagenesis and genome editing in crop improvement: perspectives for the global regulatory landscape. Trends Plant Sci. 26, 1258–1269. doi: 10.1016/j.tplants.2021.08.002, PMID: 34465535

[ref59] Kallimasioti-Pazi E. M. Thelakkad Chathoth K. Taylor G. C. Meynert A. Ballinger T. Kelder M. J. E.. (2018). Heterochromatin delays CRISPR-Cas9 mutagenesis but does not influence the outcome of mutagenic DNA repair. PLoS Biol. 16:e2005595. doi: 10.1371/journal.pbio.2005595, PMID: 30540740 PMC6306241

[ref60] Kim H. K. Kim Y. Lee S. Min S. Bae J. Y. Choi J. W.. (2019). SpCas9 activity prediction by DeepSpCas9, a deep learning–based model with high generalization performance. Sci. Adv. 5:eaax9249. doi: 10.1126/sciadv.aax9249, PMID: 31723604 PMC6834390

[ref61] Kim N. Kim H. K. Lee S. Seo J. H. Choi J. W. Park J.. (2020b). Prediction of the sequence-specific cleavage activity of Cas9 variants. Nat. Biotechnol. 38, 1328–1336. doi: 10.1038/s41587-020-0537-9, PMID: 32514125

[ref62] Kim H. Kim S.-T. Ryu J. Kang B.-C. Kim J.-S. Kim S.-G. (2017). CRISPR/Cpf1-mediated DNA-free plant genome editing. Nat. Commun. 8:14406. doi: 10.1038/ncomms14406, PMID: 28205546 PMC5316869

[ref63] Kim D. Y. Moon S. B. Ko J.-H. Kim Y.-S. Kim D. (2020a). Unbiased investigation of specificities of prime editing systems in human cells. Nucleic Acids Res. 48, 10576–10589. doi: 10.1093/nar/gkaa764, PMID: 32941652 PMC7544197

[ref64] Kim H. K. Yu G. Park J. Min S. Lee S. Yoon S.. (2021). Predicting the efficiency of prime editing guide RNAs in human cells. Nat. Biotechnol. 39, 198–206. doi: 10.1038/s41587-020-0677-y, PMID: 32958957

[ref65] Kleinstiver B. P. Prew M. S. Tsai S. Q. Topkar V. V. Nguyen N. T. Zheng Z.. (2015). Engineered CRISPR-Cas9 nucleases with altered PAM specificities. Nature 523, 481–485. doi: 10.1038/nature14592, PMID: 26098369 PMC4540238

[ref66] Kleinstiver B. P. Sousa A. A. Walton R. T. Tak Y. E. Hsu J. Y. Clement K.. (2020). Engineered CRISPR–Cas12a variants with increased activities and improved targeting ranges for gene, epigenetic and base editing. Nat. Biotechnol. 38:901. doi: 10.1038/s41587-020-0587-z, PMID: 32541959

[ref67] Koblan L. W. Doman J. L. Wilson C. Levy J. M. Tay T. Newby G. A.. (2018). Improving cytidine and adenine base editors by expression optimization and ancestral reconstruction. Nat. Biotechnol. 36, 843–846. doi: 10.1038/nbt.4172, PMID: 29813047 PMC6126947

[ref68] Komor A. C. Kim Y. B. Packer M. S. Zuris J. A. Liu D. R. (2016). Programmable editing of a target base in genomic DNA without double-stranded DNA cleavage. Nature 533, 420–424. doi: 10.1038/nature17946, PMID: 27096365 PMC4873371

[ref69] Koonin E. V. Makarova K. S. Zhang F. (2017). Diversity, classification and evolution of CRISPR-Cas systems. Curr. Opin. Microbiol. 37, 67–78. doi: 10.1016/j.mib.2017.05.008, PMID: 28605718 PMC5776717

[ref70] Kumar V. Jain M. (2015). The CRISPR–Cas system for plant genome editing: advances and opportunities. J. Exp. Bot. 66, 47–57. doi: 10.1093/jxb/eru429, PMID: 25371501

[ref71] Kunz C. Saito Y. Schär P. (2009). DNA repair in mammalian cells: mismatched repair: variations on a theme. Cell. Mol. Life Sci. 66, 1021–1038. doi: 10.1007/s00018-009-8739-9, PMID: 19153655 PMC11131451

[ref72] Kurt I. C. Zhou R. Iyer S. Garcia S. P. Miller B. R. Langner L. M.. (2021). CRISPR C-to-G base editors for inducing targeted DNA transversions in human cells. Nat. Biotechnol. 39, 41–46. doi: 10.1038/s41587-020-0609-x, PMID: 32690971 PMC7854778

[ref73] Lawrenson T. Shorinola O. Stacey N. Li C. Østergaard L. Patron N.. (2015). Induction of targeted, heritable mutations in barley and Brassica oleracea using RNA-guided Cas9 nuclease. Genome Biol. 16:258. doi: 10.1186/s13059-015-0826-7, PMID: 26616834 PMC4663725

[ref74] Lee K. Zhang Y. Kleinstiver B. P. Guo J. A. Aryee M. J. Miller J.. (2019). Activities and specificities of CRISPR/Cas9 and Cas12a nucleases for targeted mutagenesis in maize. Plant Biotechnol. J. 17, 362–372. doi: 10.1111/pbi.12982, PMID: 29972722 PMC6320322

[ref75] Lei Y. Lu L. Liu H.-Y. Li S. Xing F. Chen L.-L. (2014). CRISPR-P: a web tool for synthetic single-guide RNA design of CRISPR-system in plants. Mol. Plant 7, 1494–1496. doi: 10.1093/mp/ssu044, PMID: 24719468

[ref76] Li H. Li J. Chen J. Yan L. Xia L. (2020b). Precise modifications of both exogenous and endogenous genes in rice by prime editing. Mol. Plant 13, 671–674. doi: 10.1016/j.molp.2020.03.011, PMID: 32222486

[ref77] Li S. Li J. He Y. Xu M. Zhang J. Du W.. (2019). Precise gene replacement in rice by RNA transcript-templated homologous recombination. Nat. Biotechnol. 37, 445–450. doi: 10.1038/s41587-019-0065-7, PMID: 30886437

[ref78] Li J. Meng X. Zong Y. Chen K. Zhang H. Liu J.. (2016). Gene replacements and insertions in rice by intron targeting using CRISPR–Cas9. Nat. Plants 2:16139. doi: 10.1038/nplants.2016.139, PMID: 27618611

[ref79] Li X. Wang Y. Liu Y. Yang B. Wang X. Wei J.. (2018b). Base editing with a Cpf1–cytidine deaminase fusion. Nat. Biotechnol. 36, 324–327. doi: 10.1038/nbt.4102, PMID: 29553573

[ref80] Li G. Zhou Z. Liang L. Song Z. Hu Y. Cui J.. (2020a). Genome-wide identification and analysis of highly specific CRISPR/Cas9 editing sites in pepper (*Capsicum annuum* L.). PLoS One 15:e0244515. doi: 10.1371/journal.pone.0244515, PMID: 33373406 PMC7771699

[ref81] Li Y. Zhu J. Wu H. Liu C. Huang C. Lan J.. (2020c). Precise base editing of non-allelic acetolactate synthase genes confers sulfonylurea herbicide resistance in maize. Crop J. 8, 449–456. doi: 10.1016/j.cj.2019.10.001

[ref82] Li C. Zong Y. Wang Y. Jin S. Zhang D. Song Q.. (2018a). Expanded base editing in rice and wheat using a Cas9-adenosine deaminase fusion. Genome Biol. 19:59. doi: 10.1186/s13059-018-1443-z, PMID: 29807545 PMC5972399

[ref83] Liang Z. Chen K. Li T. Zhang Y. Wang Y. Zhao Q.. (2017). Efficient DNA-free genome editing of bread wheat using CRISPR/Cas9 ribonucleoprotein complexes. Nat. Commun. 8:14261. doi: 10.1038/ncomms14261, PMID: 28098143 PMC5253684

[ref84] Lim D. Maas W. K. (1989). Reverse transcriptase-dependent synthesis of a covalently linked, branched DNA-RNA compound in *E. coli* B. Cell 56, 891–904. doi: 10.1016/0092-8674(89)90693-4, PMID: 2466573

[ref85] Lin Q. Jin S. Zong Y. Yu H. Zhu Z. Liu G.. (2021). High-efficiency prime editing with optimized, paired pegRNAs in plants. Nat. Biotechnol. 39, 923–927. doi: 10.1038/s41587-021-00868-w, PMID: 33767395

[ref86] Lin J. Wong K.-C. (2018). Off-target predictions in CRISPR-Cas9 gene editing using deep learning. Bioinformatics 34, i656–i663. doi: 10.1093/bioinformatics/bty554, PMID: 30423072 PMC6129261

[ref87] Lin Q. Zong Y. Xue C. Wang S. Jin S. Zhu Z.. (2020). Prime genome editing in rice and wheat. Nat. Biotechnol. 38, 582–585. doi: 10.1038/s41587-020-0455-x, PMID: 32393904

[ref88] Liu L. Chen P. Wang M. Li X. Wang J. Yin M.. (2017b). C2c1-sgRNA complex structure reveals RNA-guided DNA cleavage mechanism. Mol. Cell 65, 310–322. doi: 10.1016/j.molcel.2016.11.040, PMID: 27989439

[ref89] Liu Q. Cheng X. Liu G. Li B. Liu X. (2020). Deep learning improves the ability of sgRNA off-target propensity prediction. BMC Bioinformatics 21:51. doi: 10.1186/s12859-020-3395-z, PMID: 32041517 PMC7011380

[ref90] Liu H. Ding Y. Zhou Y. Jin W. Xie K. Chen L.-L. (2017a). CRISPR-P 2.0: an improved CRISPR-Cas9 tool for genome editing in plants. Mol. Plant 10, 530–532. doi: 10.1016/j.molp.2017.01.003, PMID: 28089950

[ref91] Liu Y. Yang G. Huang S. Li X. Wang X. Li G.. (2021). Enhancing prime editing by Csy4-mediated processing of pegRNA. Cell Res. 31, 1134–1136. doi: 10.1038/s41422-021-00520-x, PMID: 34103663 PMC8486859

[ref92] Lowder L. G. Zhou J. Zhang Y. Malzahn A. Zhong Z. Hsieh T.-F.. (2018). Robust transcriptional activation in plants using multiplexed CRISPR-Act2.0 and mTALE-act systems. Mol. Plant 11, 245–256. doi: 10.1016/j.molp.2017.11.010, PMID: 29197638

[ref93] Lu Y. Tian Y. Shen R. Yao Q. Wang M. Chen M.. (2020). Targeted, efficient sequence insertion and replacement in rice. Nat. Biotechnol. 38, 1402–1407. doi: 10.1038/s41587-020-0581-5, PMID: 32632302

[ref94] Lu Y. Tian Y. Shen R. Yao Q. Zhong D. Zhang X.. (2021). Precise genome modification in tomato using an improved prime editing system. Plant Biotechnol. J. 19, 415–417. doi: 10.1111/pbi.13497, PMID: 33091225 PMC7955883

[ref95] Makarova K. S. Aravind L. Wolf Y. I. Koonin E. V. (2011a). Unification of Cas protein families and a simple scenario for the origin and evolution of CRISPR-Cas systems. Biol. Direct 6:38. doi: 10.1186/1745-6150-6-38, PMID: 21756346 PMC3150331

[ref96] Makarova K. S. Haft D. H. Barrangou R. Brouns S. J. J. Charpentier E. Horvath P.. (2011b). Evolution and classification of the CRISPR–Cas systems. Nat. Rev. Microbiol. 9, 467–477. doi: 10.1038/nrmicro2577, PMID: 21552286 PMC3380444

[ref97] Malzahn A. A. Tang X. Lee K. Ren Q. Sretenovic S. Zhang Y.. (2019). Application of CRISPR-Cas12a temperature sensitivity for improved genome editing in rice, maize, and Arabidopsis. BMC Biol. 17:9. doi: 10.1186/s12915-019-0629-5, PMID: 30704461 PMC6357469

[ref98] Meaker G. A. Hair E. J. Gorochowski T. E. (2020). Advances in engineering CRISPR-Cas9 as a molecular Swiss Army knife. Synth. Biol. 5:ysaa021. doi: 10.1093/synbio/ysaa021, PMID: 33344779 PMC7737000

[ref99] Merker L. Schindele P. Huang T.-K. Wolter F. Puchta H. (2020). Enhancing in planta gene targeting efficiencies in Arabidopsis using temperature-tolerant CRISPR/LbCas12a. Plant Biotechnol. J. 18, 2382–2384. doi: 10.1111/pbi.13426, PMID: 32473055 PMC7680533

[ref100] Miki D. Zhang W. Zeng W. Feng Z. Zhu J.-K. (2018). CRISPR/Cas9-mediated gene targeting in Arabidopsis using sequential transformation. Nat. Commun. 9:1967. doi: 10.1038/s41467-018-04416-0, PMID: 29773790 PMC5958078

[ref101] Ming M. Ren Q. Pan C. He Y. Zhang Y. Liu S.. (2020). CRISPR–Cas12b enables efficient plant genome engineering. Nat. Plants 6, 202–208. doi: 10.1038/s41477-020-0614-6, PMID: 32170285

[ref102] Minkenberg B. Zhang J. Xie K. Yang Y. (2019). CRISPR - PLANT v2: an online resource for highly specific guide RNA spacers based on improved off-target analysis. Plant Biotechnol. J. 17, 5–8. doi: 10.1111/pbi.13025, PMID: 30325102 PMC6330547

[ref103] Miyaoka Y. Berman J. R. Cooper S. B. Mayerl S. J. Chan A. H. Zhang B.. (2016). Systematic quantification of HDR and NHEJ reveals effects of locus, nuclease, and cell type on genome-editing. Sci. Rep. 6:23549. doi: 10.1038/srep23549, PMID: 27030102 PMC4814844

[ref104] Mojica F. J. M. Diez-Villasenor C. Soria E. Juez G. (2000). Biological significance of a family of regularly spaced repeats in the genomes of Archaea, bacteria and mitochondria. Mol. Microbiol. 36, 244–246. doi: 10.1046/j.1365-2958.2000.01838.x, PMID: 10760181

[ref105] Mojica F. J. M. Rodriguez-Valera F. (2016). The discovery of CRISPR in archaea and bacteria. FEBS J. 283, 3162–3169. doi: 10.1111/febs.13766, PMID: 27234458

[ref106] Moreno-Mateos M. A. Fernandez J. P. Rouet R. Vejnar C. E. Lane M. A. Mis E.. (2017). CRISPR-Cpf1 mediates efficient homology-directed repair and temperature-controlled genome editing. Nat. Commun. 8:2024. doi: 10.1038/s41467-017-01836-2, PMID: 29222508 PMC5722943

[ref107] Muringai V. Fan X. Goddard E. (2020). Canadian consumer acceptance of gene-edited versus genetically modified potatoes: a choice experiment approach. Can. J. Agric. Econ. 68, 47–63. doi: 10.1111/cjag.12221

[ref108] Nakade S. Tsubota T. Sakane Y. Kume S. Sakamoto N. Obara M.. (2014). Microhomology-mediated end-joining-dependent integration of donor DNA in cells and animals using TALENs and CRISPR/Cas9. Nat. Commun. 5:5560. doi: 10.1038/ncomms6560, PMID: 25410609 PMC4263139

[ref109] Negishi K. Mikami M. Toki S. Endo M. (2020). Enhanced FnCas12a-mediated targeted mutagenesis using crRNA With altered target length in rice. Front. Genome Ed. 2:608563. doi: 10.3389/fgeed.2020.608563, PMID: 34713233 PMC8525410

[ref110] Nishida K. Arazoe T. Yachie N. Banno S. Kakimoto M. Tabata M.. (2016). Targeted nucleotide editing using hybrid prokaryotic and vertebrate adaptive immune systems. Science 353:aaf8729. doi: 10.1126/science.aaf8729, PMID: 27492474

[ref111] Nishimasu H. Shi X. Ishiguro S. Gao L. Hirano S. Okazaki S.. (2018). Engineered CRISPR-Cas9 nuclease with expanded targeting space. Science 361, 1259–1262. doi: 10.1126/science.aas9129, PMID: 30166441 PMC6368452

[ref112] O’Brien A. R. Burgio G. Bauer D. C. (2021). Domain-specific introduction to machine learning terminology, pitfalls and opportunities in CRISPR-based gene editing. Brief. Bioinform. 22, 308–314. doi: 10.1093/bib/bbz145, PMID: 32008042 PMC7820861

[ref113] Orellana L. (2019). Large-scale conformational changes and protein function: breaking the in silico barrier. Front. Mol. Biosci. 6:117. doi: 10.3389/fmolb.2019.00117, PMID: 31750315 PMC6848229

[ref114] Osakabe Y. Osakabe K. (2015). Genome editing with engineered nucleases in plants. Plant Cell Physiol. 56, 389–400. doi: 10.1093/pcp/pcu170, PMID: 25416289

[ref115] Oz M. T. Altpeter A. Karan R. Merotto A. Altpeter F. (2021). CRISPR/Cas9-mediated multi-allelic gene targeting in sugarcane confers herbicide tolerance. Front. Genome Ed. 3:15. doi: 10.3389/fgeed.2021.673566, PMID: 34713261 PMC8525412

[ref116] Padilha V. A. Alkhnbashi O. S. Shah S. A. de Carvalho A. C. P. L. F. Backofen R. (2020). CRISPRcasIdentifier: machine learning for accurate identification and classification of CRISPR-Cas systems. GigaScience 9:giaa062. doi: 10.1093/gigascience/giaa062, PMID: 32556168 PMC7298778

[ref117] Palermo G. Chen J. S. Ricci C. G. Rivalta I. Jinek M. Batista V. S.. (2018). Key role of the REC lobe during CRISPR–Cas9 activation by ‘sensing’, ‘regulating’, and ‘locking’ the catalytic HNH domain. Q. Rev. Biophys. 51:e9. doi: 10.1017/S0033583518000070, PMID: 30555184 PMC6292676

[ref118] Palermo G. Miao Y. Walker R. C. Jinek M. McCammon J. A. (2017). CRISPR-Cas9 conformational activation as elucidated from enhanced molecular simulations. Proc. Natl. Acad. Sci. U. S. A. 114, 7260–7265. doi: 10.1073/pnas.1707645114, PMID: 28652374 PMC5514767

[ref150] Perroud F. P. Guyon-Debast A. Veillet F. Kermarrec M. P. Chauvin L. Chauvin J. E.. (2022). Prime Editing in the model plant Physcomitrium patens and its potential in the tetraploid potato. Plant Sci. 316:111162. doi: 10.1016/j.plantsci.2021.111162 35151447

[ref119] Pew Research Center (2015). Americans, Politics and Science Issues. Available at: https://www.pewresearch.org/internet/wp-content/uploads/sites/9/2015/07/2015-07-01_science-and-politics_FINAL-1.pdf (Accessed March 1, 2022).

[ref120] Plant A. L. Covey S. N. Grierson D. (1985). Detection of a subgenomic mRNA for gene V, the putative reverse transcriptase gene of cauliflower mosaic virus. Nucleic Acids Res. 13, 8305–8321. doi: 10.1093/nar/13.23.8305, PMID: 2417196 PMC322136

[ref121] Poelwijk F. J. Kiviet D. J. Weinreich D. M. Tans S. J. (2007). Empirical fitness landscapes reveal accessible evolutionary paths. Nature 445, 383–386. doi: 10.1038/nature05451, PMID: 17251971

[ref004] Qin R. Liao S. Li J. Li H. Liu X. Yang J.. (2020). Increasing fidelity and efficiency by modifying cytidine base-editing systems in rice. Crop J. 8, 396–402. doi: 10.1016/j.cj.2019.04.007, PMID: 31116473

[ref122] Qin L. Li J. Wang Q. Xu Z. Sun L. Alariqi M.. (2020a). High-efficient and precise base editing of C•G to T•A in the allotetraploid cotton *Gossypium hirsutum* genome using a modified CRISPR/Cas9 system. Plant Biotechnol. J. 18, 45–56. doi: 10.1111/pbi.13168, PMID: 31116473 PMC6920158

[ref123] Qin R. Liao S. Li J. Li H. Liu X. Yang J.. (2020b). Increasing fidelity and efficiency by modifying cytidine base-editing systems in rice. Crop J. 8, 396–402. doi: 10.1016/j.cj.2019.04.007

[ref124] Que Q. Chen Z. Kelliher T. Skibbe D. Dong S. Chilton M.-D. (2019). “Plant DNA repair pathways and their applications in genome engineering,” in Plant Genome Editing with CRISPR Systems Methods in Molecular Biology. ed. Qi Y. (New York, NY: Springer New York), 3–24. 10.1007/978-1-4939-8991-1_1 30610624

[ref125] Raitskin O. Schudoma C. West A. Patron N. J. (2019). Comparison of efficiency and specificity of CRISPR-associated (Cas) nucleases in plants: an expanded toolkit for precision genome engineering. PLoS One 14:e0211598. doi: 10.1371/journal.pone.0211598, PMID: 30811422 PMC6392405

[ref126] Rees H. A. Liu D. R. (2018). Base editing: precision chemistry on the genome and transcriptome of living cells. Nat. Rev. Genet. 19, 770–788. doi: 10.1038/s41576-018-0059-1, PMID: 30323312 PMC6535181

[ref127] Ren B. Liu L. Li S. Kuang Y. Wang J. Zhang D.. (2019a). Cas9-NG greatly expands the targeting scope of the genome-editing toolkit by recognizing NG and other atypical PAMs in rice. Mol. Plant 12, 1015–1026. doi: 10.1016/j.molp.2019.03.010, PMID: 30928635

[ref128] Ren F. Ren C. Zhang Z. Duan W. Lecourieux D. Li S.. (2019b). Efficiency optimization of CRISPR/Cas9-mediated targeted mutagenesis in grape. Front. Plant Sci. 10:612. doi: 10.3389/fpls.2019.00612, PMID: 31156675 PMC6532431

[ref129] Ren Q. Sretenovic S. Liu S. Tang X. Huang L. He Y.. (2021). PAM-less plant genome editing using a CRISPR–SpRY toolbox. Nat. Plants 7, 25–33. doi: 10.1038/s41477-020-00827-4, PMID: 33398158

[ref130] Romero P. A. Arnold F. H. (2009). Exploring protein fitness landscapes by directed evolution. Nat. Rev. Mol. Cell Biol. 10, 866–876. doi: 10.1038/nrm2805, PMID: 19935669 PMC2997618

[ref131] Safari F. Zare K. Negahdaripour M. Barekati-Mowahed M. Ghasemi Y. (2019). CRISPR Cpf1 proteins: structure, function and implications for genome editing. Cell Biosci. 9:36. doi: 10.1186/s13578-019-0298-7, PMID: 31086658 PMC6507119

[ref132] Salwan R. Sharma A. Sharma V. (2020). “CRISPR/Cas system of prokaryotic extremophiles and its applications,” in Physiological and Biotechnological Aspects of Extremophiles. eds. Salwan R. Sharma V. (London, UK: Elsevier), 155–168.

[ref133] Schindele P. Puchta H. (2020). Engineering CRISPR/Cas12a for highly efficient, temperature-tolerant plant gene editing. Plant Biotechnol. J. 18, 1118–1120. doi: 10.1111/pbi.13275, PMID: 31606929 PMC7152607

[ref134] Schindele P. Wolter F. Puchta H. (2018). Transforming plant biology and breeding with CRISPR/Cas9, Cas12 and Cas13. FEBS Lett. 592, 1954–1967. doi: 10.1002/1873-3468.13073, PMID: 29710373

[ref135] Schmid-Burgk J. L. Gao L. Li D. Gardner Z. Strecker J. Lash B.. (2020). Highly parallel profiling of Cas9 variant specificity. Mol. Cell 78, 794–800.e8. doi: 10.1016/j.molcel.2020.02.023, PMID: 32187529 PMC7370240

[ref136] Schubert M. S. Thommandru B. Woodley J. Turk R. Yan S. Kurgan G.. (2021). Optimized design parameters for CRISPR Cas9 and Cas12a homology-directed repair. Sci. Rep. 11:19482. doi: 10.1038/s41598-021-98965-y, PMID: 34593942 PMC8484621

[ref137] Shimatani Z. Kashojiya S. Takayama M. Terada R. Arazoe T. Ishii H.. (2017). Targeted base editing in rice and tomato using a CRISPR-Cas9 cytidine deaminase fusion. Nat. Biotechnol. 35, 441–443. doi: 10.1038/nbt.3833, PMID: 28346401

[ref138] Shmakov S. Abudayyeh O. O. Makarova K. S. Wolf Y. I. Gootenberg J. S. Semenova E.. (2015). Discovery and functional characterization of diverse class 2 CRISPR-Cas systems. Mol. Cell 60, 385–397. doi: 10.1016/j.molcel.2015.10.008, PMID: 26593719 PMC4660269

[ref139] Sledzinski P. Nowaczyk M. Olejniczak M. (2020). Computational tools and resources supporting CRISPR-Cas experiments. Cell 9:1288. doi: 10.3390/cells9051288, PMID: 32455882 PMC7290941

[ref140] Sretenovic S. Liu S. Li G. Cheng Y. Fan T. Xu Y.. (2021). Exploring C-To-G Base editing in Rice, tomato, and poplar. Front. Genome Ed. 3:756766. doi: 10.3389/fgeed.2021.756766, PMID: 34713268 PMC8525388

[ref141] Strecker J. Jones S. Koopal B. Schmid-Burgk J. Zetsche B. Gao L.. (2019). Engineering of CRISPR-Cas12b for human genome editing. Nat. Commun. 10:212. doi: 10.1038/s41467-018-08224-4, PMID: 30670702 PMC6342934

[ref142] Tálas A. Huszár K. Kulcsár P. I. Varga J. K. Varga É. Tóth E.. (2021). A method for characterizing Cas9 variants via a one-million target sequence library of self-targeting sgRNAs. Nucleic Acids Res. 49:e31. doi: 10.1093/nar/gkaa1220, PMID: 33450024 PMC8034649

[ref143] Tang X. Lowder L. G. Zhang T. Malzahn A. A. Zheng X. Voytas D. F.. (2017). A CRISPR–Cpf1 system for efficient genome editing and transcriptional repression in plants. Nat. Plants 3:17018. doi: 10.1038/s41477-017-0001-0, PMID: 28211909

[ref144] Tang X. Ren Q. Yang L. Bao Y. Zhong Z. He Y.. (2019). Single transcript unit CRISPR 2.0 systems for robust Cas9 and Cas12a mediated plant genome editing. Plant Biotechnol. J. 17, 1431–1445. doi: 10.1111/pbi.13068, PMID: 30582653 PMC6576101

[ref145] Tang X. Sretenovic S. Ren Q. Jia X. Li M. Fan T.. (2020). Plant prime editors enable precise gene editing in rice cells. Mol. Plant 13, 667–670. doi: 10.1016/j.molp.2020.03.010, PMID: 32222487

[ref146] Teng F. Cui T. Feng G. Guo L. Xu K. Gao Q.. (2018). Repurposing CRISPR-Cas12b for mammalian genome engineering. Cell Discov. 4:63. doi: 10.1038/s41421-018-0069-3, PMID: 30510770 PMC6255809

[ref147] Tóth E. Varga É. Kulcsár P. I. Kocsis-Jutka V. Krausz S. L. Nyeste A.. (2020). Improved LbCas12a variants with altered PAM specificities further broaden the genome targeting range of Cas12a nucleases. Nucleic Acids Res. 48, 3722–3733. doi: 10.1093/nar/gkaa110, PMID: 32107556 PMC7144938

[ref148] Truong L. N. Li Y. Shi L. Z. Hwang P. Y.-H. He J. Wang H.. (2013). Microhomology-mediated end joining and homologous recombination share the initial end resection step to repair DNA double-strand breaks in mammalian cells. Proc. Natl. Acad. Sci. 110, 7720–7725. doi: 10.1073/pnas.1213431110, PMID: 23610439 PMC3651503

[ref149] Tyagi S. Kesiraju K. Saakre M. Rathinam M. Raman V. Pattanayak D.. (2020). Genome editing for resistance to insect pests: an emerging tool for crop improvement. ACS Omega 5, 20674–20683. doi: 10.1021/acsomega.0c01435, PMID: 32875201 PMC7450494

[ref151] Verkuijl S. A. Rots M. G. (2019). The influence of eukaryotic chromatin state on CRISPR–Cas9 editing efficiencies. Curr. Opin. Biotechnol. 55, 68–73. doi: 10.1016/j.copbio.2018.07.005, PMID: 30189348

[ref152] Vu T. V. Sivankalyani V. Kim E.-J. Doan D. T. H. Tran M. T. Kim J.. (2020). Highly efficient homology-directed repair using CRISPR/Cpf1-geminiviral replicon in tomato. Plant Biotechnol. J. 18, 2133–2143. doi: 10.1111/pbi.13373, PMID: 32176419 PMC7540044

[ref153] Walton R. T. Christie K. A. Whittaker M. N. Kleinstiver B. P. (2020). Unconstrained genome targeting with near-PAMless engineered CRISPR-Cas9 variants. Science 368, 290–296. doi: 10.1126/science.aba8853, PMID: 32217751 PMC7297043

[ref154] Waltz E. (2016). Gene-edited CRISPR mushroom escapes US regulation. Nature 532:293. doi: 10.1038/nature.2016.19754, PMID: 27111611

[ref155] Wang Q. Alariqi M. Wang F. Li B. Ding X. Rui H.. (2020d). The application of a heat-inducible CRISPR/Cas12b (C2c1) genome editing system in tetraploid cotton (*G. hirsutum*) plants. Plant Biotechnol. J. 18, 2436–2443. doi: 10.1111/pbi.13417, PMID: 32438486 PMC7680538

[ref156] Wang Y. Cheng X. Shan Q. Zhang Y. Liu J. Gao C.. (2014). Simultaneous editing of three homoeoalleles in hexaploid bread wheat confers heritable resistance to powdery mildew. Nat. Biotechnol. 32, 947–951. doi: 10.1038/nbt.2969, PMID: 25038773

[ref157] Wang H. Cimen E. Singh N. Buckler E. (2020b). Deep learning for plant genomics and crop improvement. Curr. Opin. Plant Biol. 54, 34–41. doi: 10.1016/j.pbi.2019.12.010, PMID: 31986354

[ref158] Wang Q. Liu J. Janssen J. M. Le Bouteiller M. Frock R. L. Gonçalves M. A. F. V. (2021). Precise and broad scope genome editing based on high-specificity Cas9 nickases. Nucleic Acids Res. 49, 1173–1198. doi: 10.1093/nar/gkaa1236, PMID: 33398349 PMC7826261

[ref159] Wang M. Lu Y. Botella J. R. Mao Y. Hua K. Zhu J. (2017a). Gene targeting by homology-directed repair in rice using a geminivirus-based CRISPR/Cas9 system. Mol. Plant 10, 1007–1010. doi: 10.1016/j.molp.2017.03.002, PMID: 28315751

[ref160] Wang M. Mao Y. Lu Y. Tao X. Zhu J. (2017b). Multiplex gene editing in rice using the CRISPR-Cpf1 system. Mol. Plant 10, 1011–1013. doi: 10.1016/j.molp.2017.03.001, PMID: 28315752

[ref161] Wang M. Mao Y. Lu Y. Wang Z. Tao X. Zhu J.-K. (2018). Multiplex gene editing in rice with simplified CRISPR-Cpf1 and CRISPR-Cas9 systems: simplified single transcriptional unit CRISPR systems. J. Integr. Plant Biol. 60, 626–631. doi: 10.1111/jipb.12667, PMID: 29762900

[ref162] Wang J. Zhang X. Cheng L. Luo Y. (2020c). An overview and metanalysis of machine and deep learning-based CRISPR gRNA design tools. RNA Biol. 17, 13–22. doi: 10.1080/15476286.2019.1669406, PMID: 31533522 PMC6948960

[ref163] Wang D. Zhang C. Wang B. Li B. Wang Q. Liu D.. (2019). Optimized CRISPR guide RNA design for two high-fidelity Cas9 variants by deep learning. Nat. Commun. 10:4284. doi: 10.1038/s41467-019-12281-8, PMID: 31537810 PMC6753114

[ref164] Wang F. Zhang C. Xu W. Yuan S. Song J. Li L.. (2020a). Developing high-efficiency base editors by combining optimized synergistic core components with new types of nuclear localization signal peptide. Crop J. 8, 408–417. doi: 10.1016/j.cj.2020.01.003

[ref165] Wei Z. Abdelrahman M. Gao Y. Ji Z. Mishra R. Sun H.. (2021). Engineering broad-spectrum resistance to bacterial blight by CRISPR-Cas9-mediated precise homology directed repair in rice. Mol. Plant 14, 1215–1218. doi: 10.1016/j.molp.2021.05.012, PMID: 33971367

[ref166] Wolabu T. W. Park J.-J. Chen M. Cong L. Ge Y. Jiang Q.. (2020). Improving the genome editing efficiency of CRISPR/Cas9 in Arabidopsis and *Medicago truncatula*. Planta 252:15. doi: 10.1007/s00425-020-03415-0, PMID: 32642859 PMC7343739

[ref167] Woo J. W. Kim J. Kwon S. I. Corvalán C. Cho S. W. Kim H.. (2015). DNA-free genome editing in plants with preassembled CRISPR-Cas9 ribonucleoproteins. Nat. Biotechnol. 33, 1162–1164. doi: 10.1038/nbt.3389, PMID: 26479191

[ref168] Wu F. Qiao X. Zhao Y. Zhang Z. Gao Y. Shi L.. (2020). Targeted mutagenesis in *Arabidopsis thaliana* using CRISPR-Cas12b/C2c1. J. Integr. Plant Biol. 62, 1653–1658. doi: 10.1111/jipb.12944, PMID: 32396228

[ref169] Wu Y. Xu W. Wang F. Zhao S. Feng F. Song J.. (2019). Increasing cytosine base editing scope and efficiency with engineered Cas9-PmCDA1 fusions and the modified sgRNA in rice. Front. Genet. 10:379. doi: 10.3389/fgene.2019.00379, PMID: 31134125 PMC6512751

[ref170] Wunderlich S. Gatto K. A. (2015). Consumer perception of genetically modified organisms and sources of information. Adv. Nutr. 6, 842–851. doi: 10.3945/an.115.008870, PMID: 26567205 PMC4642419

[ref171] Xiang X. Corsi G. I. Anthon C. Qu K. Pan X. Liang X.. (2021). Enhancing CRISPR-Cas9 gRNA efficiency prediction by data integration and deep learning. Nat. Commun. 12:3238. doi: 10.1038/s41467-021-23576-0, PMID: 34050182 PMC8163799

[ref172] Xie K. Zhang J. Yang Y. (2014). Genome-wide prediction of highly specific guide RNA spacers for CRISPR–Cas9-mediated genome editing in model plants and major crops. Mol. Plant 7, 923–926. doi: 10.1093/mp/ssu009, PMID: 24482433

[ref173] Xu Z. Kuang Y. Ren B. Yan D. Yan F. Spetz C.. (2021). SpRY greatly expands the genome editing scope in rice with highly flexible PAM recognition. Genome Biol. 22:6. doi: 10.1186/s13059-020-02231-9, PMID: 33397431 PMC7780387

[ref174] Xu R. Li J. Liu X. Shan T. Qin R. Wei P. (2020a). Development of plant prime-editing systems for precise genome editing. Plant Commun. 1:100043. doi: 10.1016/j.xplc.2020.100043, PMID: 33367239 PMC7747961

[ref175] Xu Y. Verma D. Sheridan R. P. Liaw A. Ma J. Marshall N. M.. (2020c). Deep dive into machine learning models for protein engineering. J. Chem. Inf. Model. 60, 2773–2790. doi: 10.1021/acs.jcim.0c00073, PMID: 32250622

[ref176] Xu H. Xiao T. Chen C.-H. Li W. Meyer C. A. Wu Q.. (2015). Sequence determinants of improved CRISPR sgRNA design. Genome Res. 25, 1147–1157. doi: 10.1101/gr.191452.115, PMID: 26063738 PMC4509999

[ref177] Xu W. Zhang C. Yang Y. Zhao S. Kang G. He X.. (2020b). Versatile nucleotides substitution in plant using an improved prime editing system. Mol. Plant 13, 675–678. doi: 10.1016/j.molp.2020.03.012, PMID: 32234340

[ref178] Yamamoto A. Ishida T. Yoshimura M. Kimura Y. Sawa S. (2019). Developing heritable mutations in *Arabidopsis thaliana* using a modified CRISPR/Cas9 toolkit comprising PAM-altered Cas9 variants and gRNAs. Plant Cell Physiol. 60, 2255–2262. doi: 10.1093/pcp/pcz118, PMID: 31198958

[ref179] Yan J. Xue D. Chuai G. Gao Y. Zhang G. Liu Q. (2020). Benchmarking and integrating genome-wide CRISPR off-target detection and prediction. Nucleic Acids Res. 48, 11370–11379. doi: 10.1093/nar/gkaa930, PMID: 33137817 PMC7672467

[ref180] Yang H. Gao P. Rajashankar K. R. Patel D. J. (2016). PAM-dependent target DNA recognition and cleavage by C2c1 CRISPR-Cas endonuclease. Cell 167, 1814–1828.e12. doi: 10.1016/j.cell.2016.11.053, PMID: 27984729 PMC5278635

[ref181] Yang M. Peng S. Sun R. Lin J. Wang N. Chen C. (2018). The conformational dynamics of Cas9 governing DNA cleavage are revealed by single-molecule FRET. Cell Rep. 22, 372–382. doi: 10.1016/j.celrep.2017.12.048, PMID: 29320734

[ref182] Yourik P. Fuchs R. T. Mabuchi M. Curcuru J. L. Robb G. B. (2019). *Staphylococcus aureus* Cas9 is a multiple-turnover enzyme. RNA 25, 35–44. doi: 10.1261/rna.067355.118, PMID: 30348755 PMC6298560

[ref183] Zafra M. P. Schatoff E. M. Katti A. Foronda M. Breinig M. Schweitzer A. Y.. (2018). Optimized base editors enable efficient editing in cells, organoids and mice. Nat. Biotechnol. 36, 888–893. doi: 10.1038/nbt.4194, PMID: 29969439 PMC6130889

[ref184] Zeng D. Li X. Huang J. Li Y. Cai S. Yu W.. (2020). Engineered Cas9 variant tools expand targeting scope of genome and base editing in rice. Plant Biotechnol. J. 18, 1348–1350. doi: 10.1111/pbi.13293, PMID: 31696609 PMC7206991

[ref185] Zetsche B. Abudayyeh O. O. Gootenberg J. S. Scott D. A. Zhang F. (2020). A survey of genome editing activity for 16 Cas12a orthologs. Keio J. Med. 69, 59–65. doi: 10.2302/kjm.2019-0009-OA, PMID: 31723075 PMC7220826

[ref186] Zetsche B. Gootenberg J. S. Abudayyeh O. O. Slaymaker I. M. Makarova K. S. Essletzbichler P.. (2015). Cpf1 is a single RNA-guided endonuclease of a class 2 CRISPR-Cas system. Cell 163, 759–771. doi: 10.1016/j.cell.2015.09.038, PMID: 26422227 PMC4638220

[ref187] Zhang G. Dai Z. Dai X. (2020). C-RNNCrispr: prediction of CRISPR/Cas9 sgRNA activity using convolutional and recurrent neural networks. Comput. Struct. Biotechnol. J. 18, 344–354. doi: 10.1016/j.csbj.2020.01.013, PMID: 32123556 PMC7037582

[ref188] Zhang Y. Liang Z. Zong Y. Wang Y. Liu J. Chen K.. (2016). Efficient and transgene-free genome editing in wheat through transient expression of CRISPR/Cas9 DNA or RNA. Nat. Commun. 7:12617. doi: 10.1038/ncomms12617, PMID: 27558837 PMC5007326

[ref189] Zhang Y. Ren Q. Tang X. Liu S. Malzahn A. A. Zhou J.. (2021). Expanding the scope of plant genome engineering with Cas12a orthologs and highly multiplexable editing systems. Nat. Commun. 12:1944. doi: 10.1038/s41467-021-22330-w, PMID: 33782402 PMC8007695

[ref190] Zhao D. Li J. Li S. Xin X. Hu M. Price M. A.. (2021). Glycosylase base editors enable C-to-A and C-to-G base changes. Nat. Biotechnol. 39, 35–40. doi: 10.1038/s41587-020-0592-2, PMID: 32690970

[ref191] Zhong Z. Sretenovic S. Ren Q. Yang L. Bao Y. Qi C.. (2019). Improving plant genome editing with high-fidelity xCas9 and non-canonical PAM-targeting Cas9-NG. Mol. Plant 12, 1027–1036. doi: 10.1016/j.molp.2019.03.011, PMID: 30928637

[ref192] Zhong Z. Zhang Y. You Q. Tang X. Ren Q. Liu S.. (2018). Plant genome editing using FnCpf1 and LbCpf1 nucleases at redefined and altered PAM sites. Mol. Plant 11, 999–1002. doi: 10.1016/j.molp.2018.03.008, PMID: 29567452

[ref193] Zischewski J. Fischer R. Bortesi L. (2017). Detection of on-target and off-target mutations generated by CRISPR/Cas9 and other sequence-specific nucleases. Biotechnol. Adv. 35, 95–104. doi: 10.1016/j.biotechadv.2016.12.003, PMID: 28011075

[ref194] Zong Y. Wang Y. Li C. Zhang R. Chen K. Ran Y.. (2017). Precise base editing in rice, wheat and maize with a Cas9-cytidine deaminase fusion. Nat. Biotechnol. 35, 438–440. doi: 10.1038/nbt.3811, PMID: 28244994

